# High-fat diet promotes prostate cancer metastasis via RPS27

**DOI:** 10.1186/s40170-024-00333-7

**Published:** 2024-02-16

**Authors:** Dameng Li, Xueying Zhou, Wenxian Xu, Yongxin Cai, Chenglong Mu, Xinchun Zhao, Tingting Tang, Chen Liang, Tao Yang, Junnian Zheng, Liang Wei, Bo Ma

**Affiliations:** 1 Cancer Institute, Xuzhou Medical University, 209 Tongshan Road, Xuzhou, 221004 Jiangsu China; 2 Center of Clinical Oncology, The Affiliated Hospital of Xuzhou Medical University, 99 West Huaihai Road, Xuzhou, 221002 Jiangsu China; 3 Jiangsu Center for the Collaboration and Innovation of Cancer Biotherapy, Xuzhou Medical University, 209 Tongshan Road, Xuzhou, 221004 Jiangsu China

**Keywords:** Prostate cancer, RPS27, High fat diet, Metastasis, Obesity

## Abstract

**Background:**

Metastasis is the leading cause of death among prostate cancer (PCa) patients. Obesity is associated with both PCa-specific and all-cause mortality. High-fat diet (HFD) is a risk factor contributing to obesity. However, the association of HFD with PCa metastasis and its underlying mechanisms are unclear.

**Methods:**

Tumor xenografts were conducted by intrasplenic injections. The ability of migration or invasion was detected by transwell assay. The expression levels of RPS27 were detected by QRT-PCR and western blot.

**Results:**

The present study verified the increase in PCa metastasis caused by HFD in mice. Bioinformatics analysis demonstrated increased RPS27 in the experimentally induced PCa in HFD mice, indicating that it is an unfavorable prognostic factor. Intrasplenic injections were used to demonstrate that RPS27 overexpression promotes, while RPS27 knockdown significantly reduces, PCa liver metastasis. Moreover, RPS27 inhibition suppresses the effects of HFD on PCa metastasis. Further mRNA sequencing analysis revealed that RPS27 promotes PCa metastasis by selectively enhancing the expression of various genes.

**Conclusion:**

Our findings indicate that HFD increases the risk of PCa metastasis by elevating RPS27 expression and, subsequently, the expression of genes involved in PRAD progression. Therefore, RPS27 may serve as a novel target for the diagnosis and treatment of metastatic PCa.

**Supplementary Information:**

The online version contains supplementary material available at 10.1186/s40170-024-00333-7.

## Introduction

Prostate cancer (PCa) is a leading cause of cancer-related deaths among men [1]. Previous studies have reported geographic differences in the incidence and mortality of PCa, and that men from low-incidence areas become more susceptible to PCa if they migrate to high-incidence areas [2]. Interestingly, the incidence and progression of PCa are correlated with the adoption of Western eating habits in these regions [3]. These studies have suggested that diet significantly influences PCa occurrence.

Typical Western diet is high in fats and dietary fat has been linked with an increased risk of PCa [4]. An increasing number of studies have shown that high fat diet (HFD) promotes PCa development. Huang et al. reported that HFD stimulates PCa cell growth and invasion by upregulating MIC-1 signaling [5]. Labbé et al. reported that HFD leads to increased cellular proliferation and tumor burden by enhancing MYC transcription [6]. A study by Fujita et al. found that HFD accelerated PCa growth by inducing IL6-mediated inflammation [7]. These studies focus on the PCa growth or invasion. However, clinical retrospective analysis shows that PCa mortality predominantly results from metastasis. Once clinically evident metastases occur, the 5-year survival rate of PCa patients plummets from nearly 100% for local and regional tumors to 30% [8]. However, the association of HFD with PCa metastasis and the underlying mechanisms have not been investigated.

The ribosomal protein RPS27, also known as metallopanstimulin-1 (MPS-1), is a member of the S27E family of ribosomal proteins [9]. Its expression is elevated in a variety of tumors, including liver cancer, gastric cancer, and glioblastoma [10–13]. Studies have found that RPS27 can enter the extracellular space through secretion or gradient diffusion, and is then released into the circulatory system. Its levels can be used as an early detection or prognostic indicator for tumors [9]. In addition to play an important role in ribosome biogenesis and protein translation, ribosomal proteins can also affect tumorigenesis and development through extra-ribosomal functions. Recent studies have reported that RPS27 causes the occurrence and development of tumors by affecting the JNK/c-JUN, NF-kB, and other signaling pathways [11, 13, 14]. However, the specific regulatory mechanisms of RPS27 in PCa have not been explored. The present study found that HFD promotes PCa metastasis, and that RPS27 plays an important role in this process.

## Materials and methods

### Mice

All animal procedures were performed in accordance with the guidelines of the institutional animal care and use committee of Xuzhou Medical University. Ethical approval for research involving animals has been obtained (IACUC Issue No. 202212S003).

NCG male mice were purchased from GemPharmatech (strain No. T001475, Nanjing, China). The mice were housed in specific-pathogen-free facilities under a 12/12-h light–dark cycle. The mice were 4–6 weeks old, and were fed a chow diet (CD) or HFD (60% kcal% fat) for 8–12 weeks.

To construct the human prostate cancer xenograft model, 5 × 10^5^ DU145 cells in 50 μL of serum-free medium were injected into the spleen [15]. The mice were euthanized four or five weeks after tumor implantation.

### Cell migration and invasion

Prostate cancer cell lines (PC3 and DU145) were obtained from the National Collection of Authenticated Cell Cultures (Shanghai, China) and identified by STR. PC3 and DU145 were maintained in 1640 medium and Dulbecco’s Modified Eagle Medium (DMEM) supplemented with 10% fetal bovine serum (FBS). The cells were cultured in a humidified incubator with 5% CO_2_ at 37°C.

The migration or invasion capacity of the cells was measured using a transwell insert containing polycarbonate filters with 8-μm pores. For invasion assay, the upper chamber surface was coated with 50 μL of 1 mg/mL matrigel matrix (Corning, 356,234).

### Establishment of RPS27 overexpression and knockdown cell lines

For over-expression, the human RPS27 cDNA were cloned into vector pLVX-IRES-ZsGreen1. For gene knockdown, the oligos containing RPS27 shRNA sequence or scramble shRNA sequence flanked by sequences that are compatible with the sticky ends of EcoRI and AgeI were annealed and ligated into vector pLKO.1. To produce lentiviral particles, the over-expression vector or the pLKO.1 vector was used with packaging plasmid psPAX2 and envelope plasmid pMD2. G. Harvest media containing lentiviral particles from 293 T cells and infect target cells. RPS27 over-expression cells stably expressing ZsGreen were sorted by flow cytometry. Cells were stably expressing shRNA were selected via addition of puromycin. The target shRNA sequences and scrambled shRNA sequence are listed in Table S2.

### Western blot

The tissues and cells were lysed using the radio immunoprecipitation assay (RIPA) buffer (Beyotime; P0013B), with the addition of protease and phosphatase inhibitor cocktails (MCE: HY-K0010, HY-K0021, and HY-K0022). The total protein concentration was measured using a BCA protein assay kit (Thermo Fisher Scientific; 23225). The primary antibodies, including RPS27 (Proteintech; 15355–1-AP), GAPDH (Proteintech; 60004–1-Ig), E-cadherin (CST; 3195), Vimentin (CST; 5741), and ECL reagents (Thermo Fisher Scientific, 34580), were used for developing the blots on nitrocellulose membranes (Cytiva, 10600002).

### Quantitative real-time polymerase chain reaction (QRT-PCR)

Total RNA was isolated from the cells or tissues using Trizol (15,596–018; Invitrogen) according to the manufacturer’s protocol. The primers for RPS27, E-cadherin, and vimentin were synthesized by Sangon Biotech (Shanghai, China). Complementary DNA (cDNA) were generated using Hiscript qRT SuperMix with gDNA wiper (Vazyme, Nanjing, China). The primer sequences are presented in Table S1. QRT-PCR was performed using LightCycler 480 II system (Roche, Mannheim, Germany). Relative mRNA expression levels were estimated using the 2-ΔΔCt method. GAPDH was used for normalization.

### Bioinformatics analysis

The RNA-seq data GSE90912 was downloaded from the NCBI GEO database (https://www.ncbi.nlm.nih.gov/geo/query/acc.cgi?acc=GSE90912), and analyzed using the IDEP online software.

### Statistical analyses

Data were presented as means ± standard error (SE). The significance of mean differences was determined using two-tailed student’s* t* test or analysis of variance (ANOVA). Statistical analyses were performed using GraphPad Prism 8.0 software (* *P* < 0.05; ** *P* < 0.01; *** *P* < 0.001).

## Results

### HFD enhances liver metastasis in intrasplenic xenograft mouse model

**Fig. 1:**
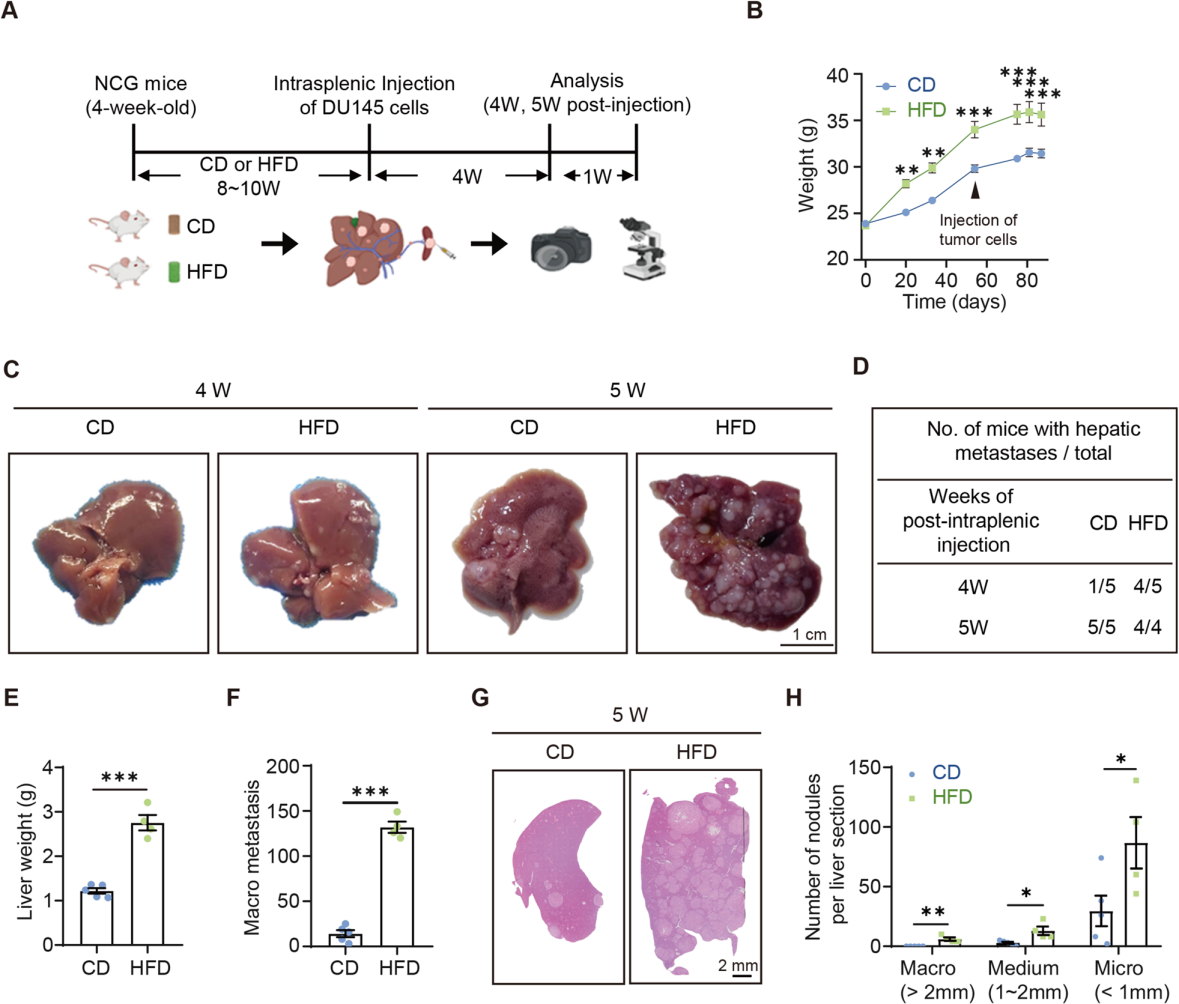
High-fat diet promoted PCa metastasis. **A** Mouse model of prostate cancer spleen-liver metastasis; **B** Body weights of CD and HFD mice (CD: chow diet; HFD: high-fat diet, D12492, research diet); **C** Representative images of liver tissue taken from CD and HFD mice; **D** Probability of liver metastasis; **E** Liver weights of CD and HFD mice after 5 weeks of inoculation; **F** Number of macro-metastatic liver tumor nodules in CD and HFD mice; **G** Representative images of hematoxylin and eosin staining of the liver tissues of CD and HFD mice 5 weeks after inoculation; **H** Number of metastatic liver tumor nodules with different sizes per section; Macro-metastases: diameter > 2 mm; Medium-metastases: 1 mm < diameter < 2 mm; Micro-metastases: diameter < 1 mm; *P < 0.05; **P < 0.01; ***P < 0.001

To explore the effect of HFD on the development of prostate cancer, an established mouse model of intrasplenic injection of human prostate cancer cells for liver metastasis was used in HFD-induced obesity mice (Fig. 1A). The weight of HFD-NCG mice was significantly greater than that of CD-NCG mice (Fig. 1B). After ectopic inoculation of PCa DU145 cells in the spleen, hepatic metastases occurred in one of the five CD mice and four of the five HFD mice at 4 weeks. All mice developed liver metastases at 5 weeks, with significantly greater liver weights and number of hepatic tumor nodules in HFD mice than in CD mice (Fig. 1C-F; Fig. S1). Hematoxylin and eosin staining revealed more metastatic tumor nodules in the livers of HFD mice, with a greater number of all stages of metastases (Fig. 1G, H; Fig. S2). We also examined the expression of epithelial-mesenchymal transition markers in the orthotopic tumors in mouse spleens. Compared to the CD mice, the tumors in HFD mice had increased vimentin and decreased E-cadherin levels (Fig. S3). These results suggest that HFD-induced obesity promotes PCa metastasis.

### HFD induces RPS27 elevation in experimental PCa models

**Fig. 2:**
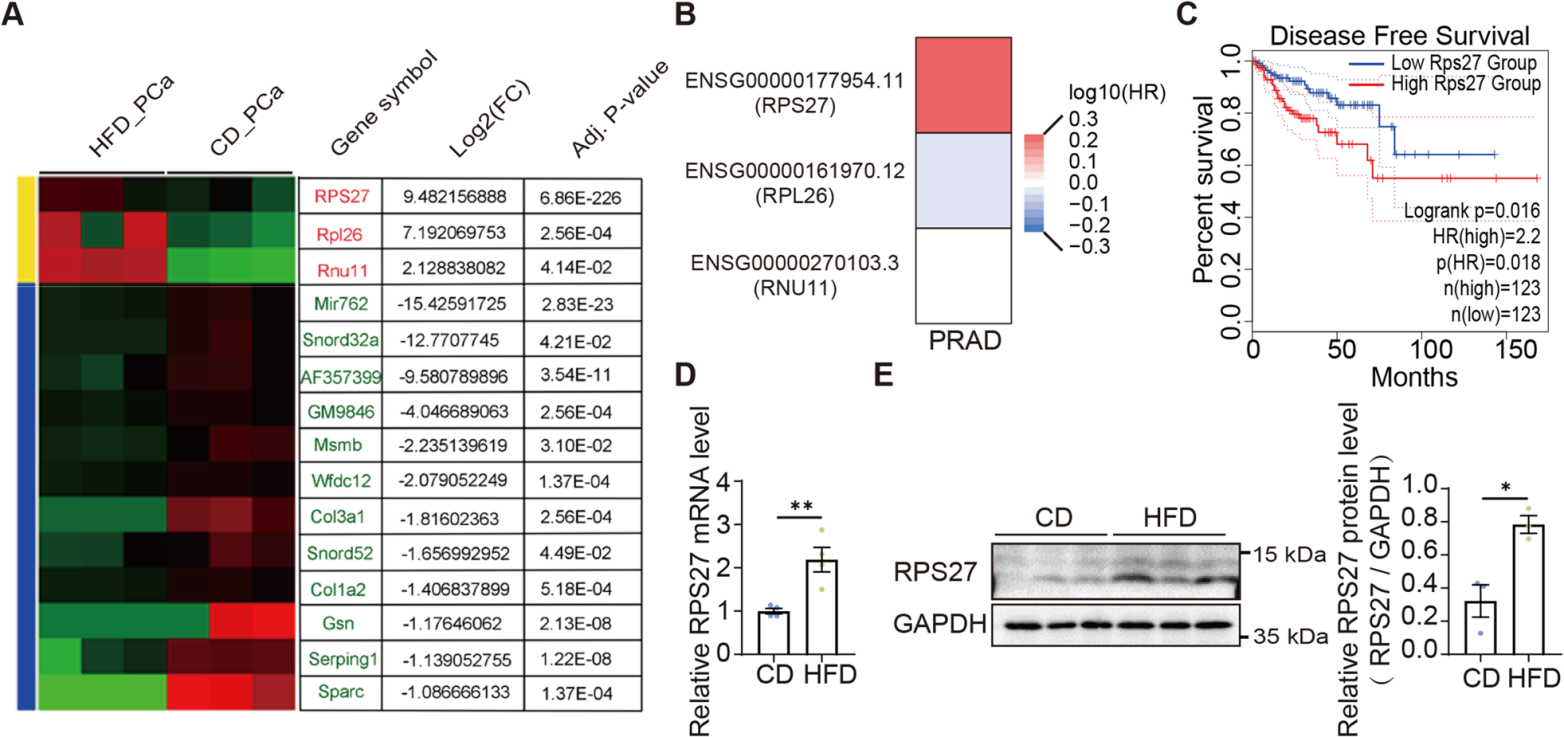
Increased RPS27 expressed in prostate cancer in HFD mice. **A** The dataset GSE90912 was analyzed to identify differentially expressed genes in prostate tumors in CD and HFD mice; Dataset we used here is part of GSE90905 (a subseries of superseries GSE90912) including GSM2417319, GSM2417320, GSM2417321, GSM2417325, GSM2417326, GSM2417327. This dataset is an expression profiling by RNA-Seq for PCa model mice fed CD or HFD [6]. **B**, **C** Survival map of up-regulated genes in PCa (**B**) and Kaplan–Meier analysis of the correlation between RPS27 expression and disease-free survival in prostate adenocarcinoma patients (**C**) via GEPIA2; Group cutoff: quartile; Datasets selection: PRAD; **D** mRNA expression level and **E** protein level of RPS27 in splenic orthotopic tumors; *P < 0.05; **P < 0.01; ***P < 0.001

To investigate the mechanism by which HFD promotes PCa metastasis, we analyzed the relevant dataset in GEO (GSE90912) and found that a series of genes were differentially expressed in experimentally induced PCa in HFD and CD mice (Fig. 2A). Interestingly, Rps27, Rpl26, and Rnu11, the first two being ribosomal proteins, were increased in HFD mice. Kaplan–Meier estimation analysis revealed that high Rps27 expression correlated with a poor prognosis in PCa patients, while Rpl26 and Rnu11 did not show any such correlations (Fig. 2B, C). In our model, RPS27 was upregulated in splenic orthotopic tumors at both mRNA (2.19 fold) and protein (2.43 fold) levels (Fig. 2D, E).

### RPS27 affects the metastatic potential of PCa cells

**Fig. 3:**
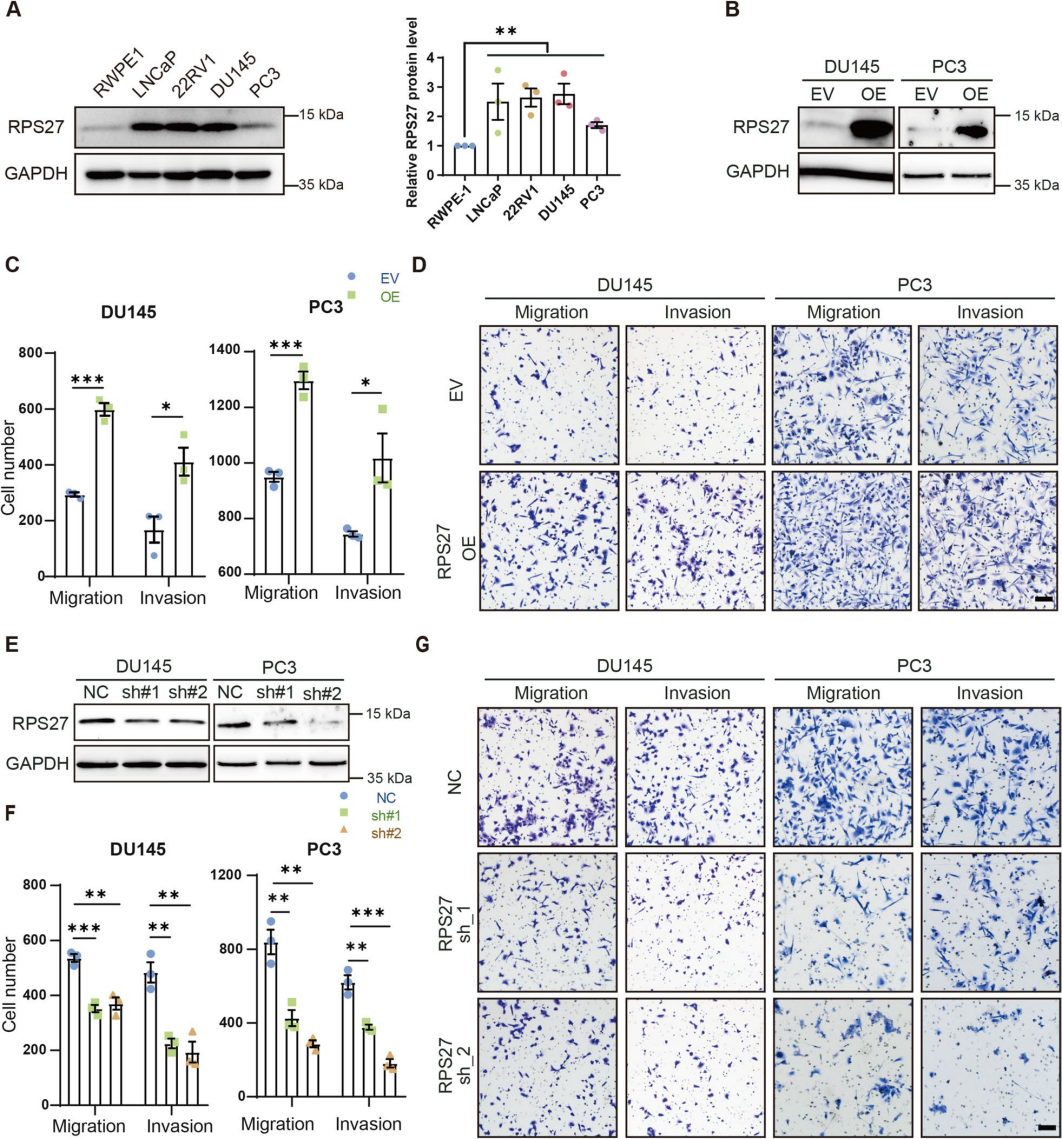
RPS27 enhanced PCa cell motility. **A** Western blot of RPS27 in normal prostate epithelial cells RWPE1 and various prostate cancer cells (LNCaP, 22RV1, DU145, and PC3); **B** Western blotting was used to detect the efficiency of RPS27 overexpression in DU145 and PC3; **C-D** Migration and invasion assay of control (EV) and RPS27-overexpressing (RPS27 OE) cells; Bar: 100 μm; (**C**) Number of cells undergoing transmembrane movement, data shown as means ± SEM for each group from three independent experiments; (**D**) Representative images; **E** Western blotting to detect the efficiency of RPS27 gene knockdown in DU145 and PC3; **F-G** Migration and invasion assay of control (NC) and RPS27 knockdown (RPS27_sh) cells; Bar: 100 μm; (**F**) Number of cells undergoing transmembrane movement, data shown as means ± SEM for each group from three independent experiments; (**G**) Representative images. *P < 0.05; **P < 0.01; ***P < 0.001

Next, the effect of altered RPS27 expression on PCa cells was investigated in vitro. RPS27 expression levels were assessed in various prostate tumor cell lines. Compared to normal prostate epithelial cells, RWPE-1, all assessed PCa cell lines had higher RPS27 expression levels (Fig. 3A; Fig. S4). Two classical PCa cell lines, DU145 and PC3, were selected for stable RPS27 overexpression (Fig. 3B), followed by migration and invasion assays. RPS27 overexpression significantly enhanced the migratory and invasive abilities of DU145 and PC3 cells (Fig. 3C, D; Fig S5). Furthermore, stable knockdown of RPS27 in DU145 and PC3 cells significantly reduced the migration and invasion (Fig. 3E-G; Fig S6). These data suggest a role of RPS27 in cell motility, and consequently in cancer metastasis.

**Fig. 4:**
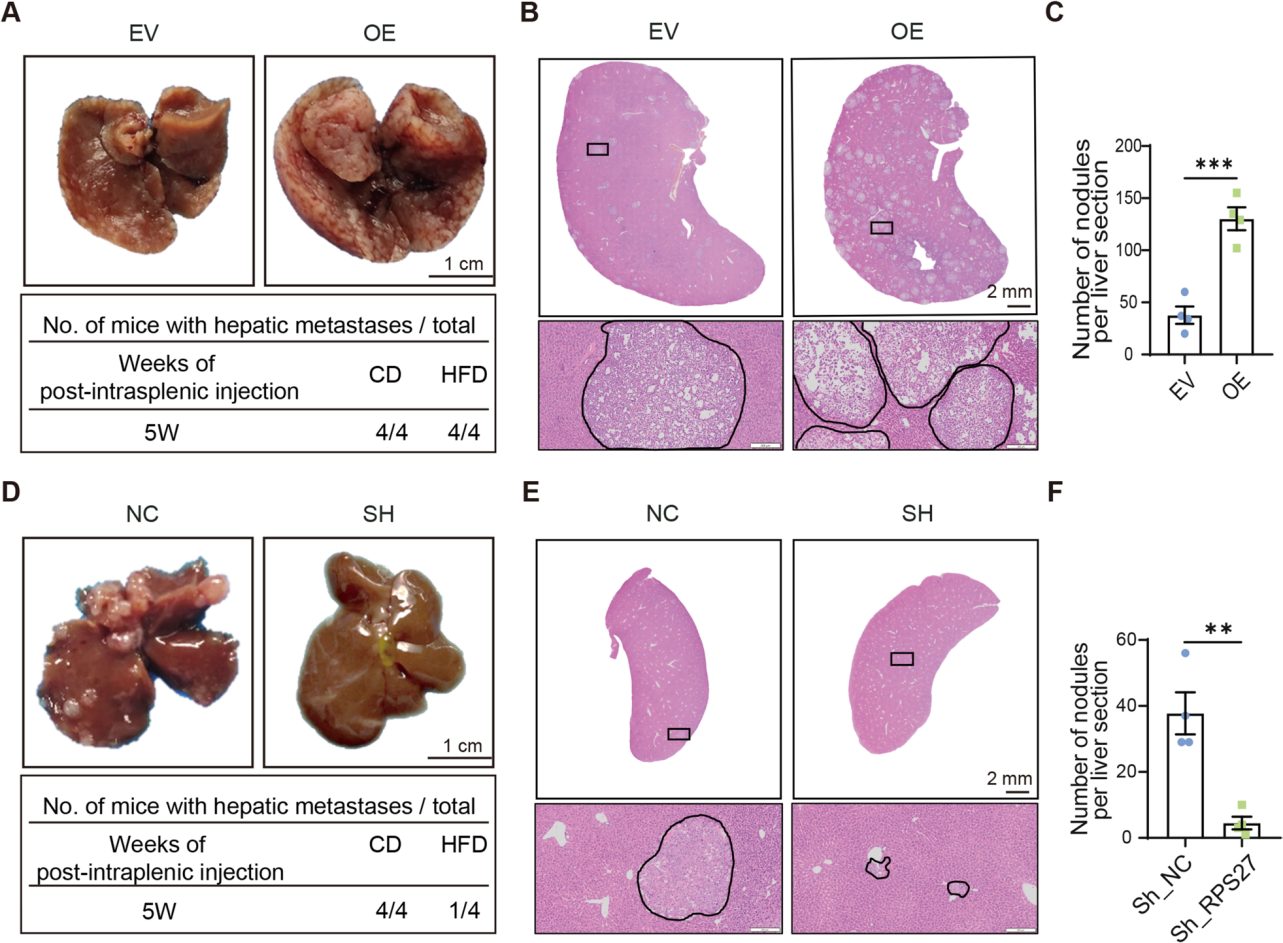
Overexpression or knockdown RPS27 affects PCa liver metastasis in vivo. **A** Representative images of liver tissues taken from EV and RPS27-OE groups; **B** Representative hematoxylin and eosin stained images of the liver tissues from EV and RPS27-OE groups; **C** Number of metastatic tumor nodules per liver section/mouse, n = 4; **D** Representative images of liver tissues taken from NC and RPS27_sh groups; **E** Representative hematoxylin and eosin stained images of the liver tissues from NC and RPS27_sh groups; **F** Number of metastatic tumor nodules per liver section/mouse, n = 4; **P < 0.01; ***P < 0.001

To verify the role of RPS27 in cancer metastasis, DU145 cells with stable RPS27 overexpression (OE) were injected into the spleens of NCG mice, while empty vector derived (EV) cells were used as controls. Both groups developed liver metastases at 5 weeks. Hematoxylin and eosin staining revealed a dramatic increase in the number of metastatic tumor nodules in the livers of mice inoculated with RPS27-OE-DU145 cells compared to those inoculated with EV-DU145 cells (Fig. 4A-C; Fig S7). Moreover, the metastatic potential of RPS27 knockdown DU145 cells was also assessed in vivo. The mice inoculated with RPS27 knockdown DU145 cells displayed fewer metastatic foci in the liver compared to the mice inoculated with control cells (Fig. 4D-F; Fig S8). Therefore, genetically manipulated expression of RPS27 may affect the metastatic potential of DU145 PCa cells, indicating its role in PCa metastasis.

### RPS27-mediated HFD-induced PCa metastasis

**Fig. 5:**
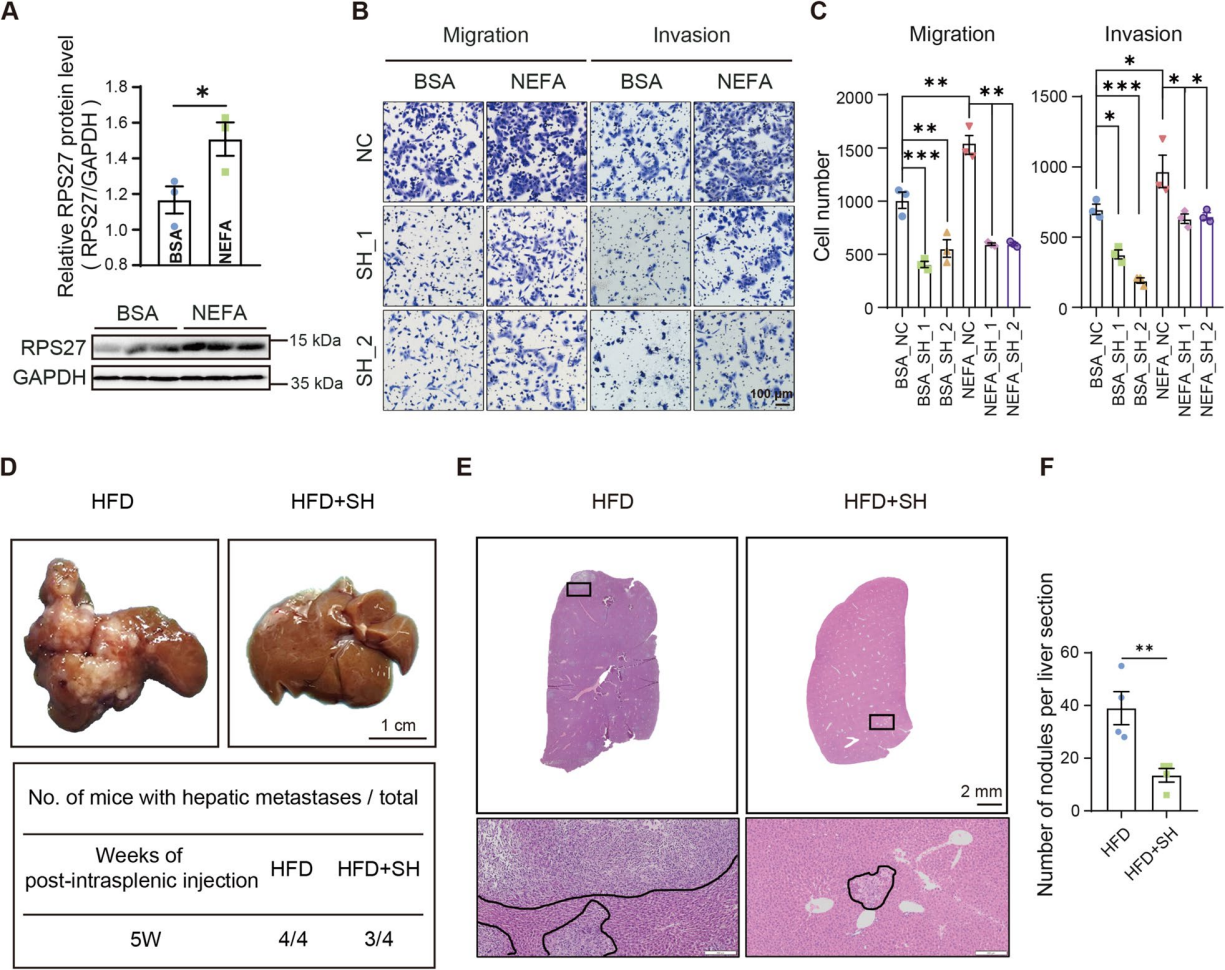
RPS27 mediates HFD-induced PCa metastasis **A** DU145 cells were treated with NEFA (50 μM OA + 50 μM PA) or BSA for 24 h, followed by Western blotting to detect RPS27 expression and quantification of RPS27 levels using ImageJ (right panel); **B** Migration and invasion assay of NEFA or BSA incubated DU145 cells; **C** Number of cells undergoing transmembrane movement, data shown as means ± SEM for each group from three independent experiments. Left panel: migration, right panel: invasion; **D** Representative images of liver tissues from HFD mice inoculated with control or RPS27_sh DU145 cells; **E** Representative hematoxylin and eosin stained images of the liver tissues; **F** Number of metastatic tumor nodules per liver section/mouse, n = 4; *P < 0.05; **P < 0.01; ***P < 0.001

To simulate the high fat environment in HFD mice in vitro, the cells were incubated with a mixture of non-esterified fatty acids (NEFA) with palmitic acid (PA) and oleic acid (OA), which led to an increased expression of RPS27 in DU145 cells (Fig. 5A). Maintenance of the DU145 cells in a NEFA-supplemented medium enhanced their invasion and migration, whereas RPS27 knockdown nullified this effect (Fig. 5B, C; Fig S9). Meanwhile, an important finding was that RPS27 knockdown prevented the HFD-induced liver metastasis of PCa cells in the mouse model. The risk of liver metastasis decreased slightly in the mice inoculated with RPS27 knockdown DU145 cells compared to those inoculated with the control cells (3/4 vs. 4/4) (Fig. 5D; Fig S10). However, the suppression of RPS27 expression significantly reduced the number of metastatic foci in the livers of HFD mice (Fig. 5E, F; Fig S10).

### RPS27 promotes prostate cancer metastasis through affecting the expression of various genes

**Fig. 6:**
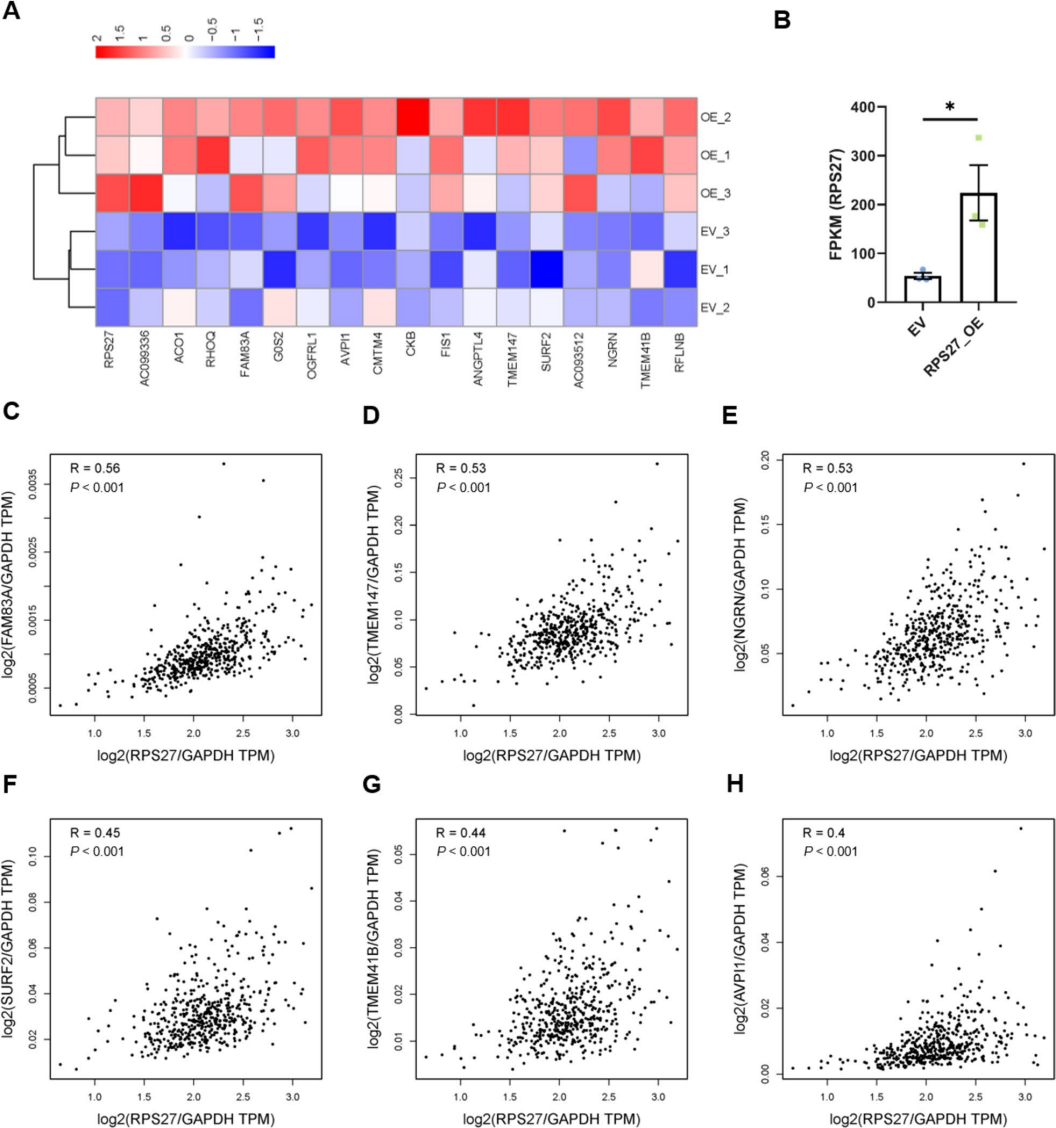
RPS27 promotes prostate cancer metastasis through affecting the expression of various genes. **A** Transcriptome sequencing was performed in DU145 cells transfected with RPS27_OE vector (OE) and empty vector (EV) to identify elevated genes in OE groups; **B** The expression level of RPS27 in OE and EV groups; C-H Genes were positively correlated with RPS27 in PRAD tumor; Analysis was performed by GEPIA2; Correlation coefficient: Pearson; (**C**) The correlation between RPS27 and FAM83A; (**D**) The correlation between RPS27 and tmem147; (**E**) The correlation between RPS27 and NGRN; (**F**) The correlation between RPS27 and SURF2; (**G**) The correlation between RPS27 and TMEM41B; (**H**) The correlation between RPS27 and AVPI1; *P < 0.05

To further explore the mechanism underlying RPS27 promoting prostate cancer cell metastasis, RNAseq was performed on DU145 cells transfected with RPS27_OE and EV (Fig. 6A). The results showed that RPS27 was significantly increased (Fig. 6B). Consistent with previous studies, we also found enhanced expression of multiple genes in RPS27_OE DU145 cells (Fig. 6A). Moreover, the relationship between RPS27 and the selected genes was analyzed and found that they were significantly correlated positively (*P* < 0.01) (Fig. 6C-H). These results indicated that RPS27 might promote PCa metastasis by increasing the expression levels of these genes.

Then we validated the correlation between RPS27 and these genes in DU145 cells by RPS27 knockdown or overexpression (Fig S12). We found that SURF2 and FAM83A were significantly elevated in RPS27_OE cells, whereas SURF2 and AVPI2 decreased in RPS27 knockdown cells. We also analyzed the correlations between patient disease-free survival and the expression of these genes. Only high expression of SURF2 is associated with poor prognosis in PRAD patients (Fig S13). These results suggested that SURF2 may be downstream of RPS27.

## Discussion

PCa is the most common cause of new cancer cases and the second most common cause of deaths among men worldwide [1]. Most of these deaths are a result of PCa metastasis [16]. The prognosis of metastatic PCa remains poor despite the various therapeutic strategies [17–20]. An investigation into the risk factors for PCa metastasis and the underlying mechanisms may guide the prevention and therapeutic strategies. Obesity, which is mainly caused by HFD, is a strong risk factor for advanced PCa. It has been reported that HFD promotes PCa growth by inducing IL6-mediated inflammation [7]. Labbé et al. found that HFD increases cell proliferation and tumor burden by enhancing MYC transcription in PCa [6]. Several studies have reported the relationship between HFD and PCa growth, but the association of HFD with PCa metastasis remains poorly understood. The present study demonstrated that HFD also promotes metastasis in PCa (Fig. 1).

In this study, we found that the expression level of RPS27 was significantly elevated in the PCa of HFD mice (Fig. 2A, D, E). The correlation analysis showed an association between high RPS27 expression and PCa recurrence (Fig. 2C). Elevated RPS27 levels have previously been observed in various cancer types, including breast cancer [10], gastric cancer [13], colorectal cancer [11], and liver cancer [21], and are associated with a poorer prognosis. However, the role of RPS27 in PCa remains unknown. Our data indicates that increased RPS27 enhances the migration and invasion abilities of PCa cells in vitro, and promotes PCa metastasis in vivo (Fig. 3C, D, Fig. 4A-C). Moreover, RPS27 inhibition could suppress the effect of HFD on PCa metastasis (Fig. 3F, G; Fig. 4D–F). These results demonstrate that RPS27 is a key regulator of HFD-promoted PCa metastasis.

As a ribosomal protein, RPS27 plays an important role in ribosome biogenesis and protein translation. Remarkably, it can regulate cellular functions via non-classical pathways. It has been reported that RPS27 and its analog RPS27L can bind to MDM2 and protect p53 from MDM2-mediated ubiquitination degradation [22, 23]. In colorectal cancers, RPS27 promotes cell proliferation by activating the JNK/c-Jun signaling pathway [11]. Besides, Ebright et al. reported that ribosomal protein (RPL15) overexpression could promote cancer metastasis by selectively enhancing the expression of some genes [24]. In this study, we also found that RPS27 overexpression could enhance the expression of some genes, including FAM83A, TMEM147, NGRN, SURF2, TMEM41B, and AVPI1 (Fig. 6A). RPS27 was also found to be positively correlated with these genes in prostate adenocarcinoma patients (Fig. 6C). Among these genes, FAM83A has been reported to be associated with the malignant behavior of various cancers [25–28]. AVPI1 has also been reported to be correlated with the invasiveness of melanoma cells [29]. Similarly, TMEM147 and TMEM41B are transmembrane proteins associated with the pathogenesis of various cancers [30–32]. SURFs were also reported to be involved in lipid homeostasis and cancers [33–36]. Therefore, we speculate that RPS27 promotes PCa metastasis by enhancing the expression of these genes.

There were some limitations of this study. First, the mechanism underlying HFD-induced RPS27 elevation in PCa was not investigated. Nonetheless, RPS27 elevation was observed at both mRNA and protein levels (Fig. 2D, E). In fact, HFD could induce various important transcription factors (TFs) and these factors maybe associated with the expression of RPS27. Through searching literatures, we found nine transcription factors induced by NEFA, including PGC1A [37], PGC1B [38], NFKB1 [39], NFKB2 [39], PPARs [40], PPRC1 [41], FOXO3 [42], MYC [43], YAP [44], E2F1 [45] and E2F2 [45]. To investigate the correlations between RPS27 and these genes, we conducted correlation analysis in PRAD tumors using GEPIA2. We found four genes exhibited moderate correlations with RPS27 (PPRC1 R = 0.54, E2F2 R = 0.52, PGC1B R = 0.51, NFKB1 R = 0.5), eight genes showed weak correlations with RPS27 (NFKB2 R = 0.47, PPARD R = 0.46, PPARA R = 0.4, MYC R = 0.38, YAP1 R = 0.35, FOXO3 R = 0.35, PGC1A R = 0.32, E2F2 R = 0.3) and an irrelevant gene (PPARG R = 0.19) (Fig. S11A). To investigate the TF(s) regulating RPS27 in PCa, we analyzed RPS27 expression level in TFs knockdown prostate cell lines using KnockTF2.0. It was found that RPS27 was decreased (Log2FC = -1.04693) in E2F2 knockdown PCa cells (method: siRNA) (Fig. S11B). Disease free survival analysis showed that E2F2 is associated with poor prognosis in PRAD (*P* = 0.00065) (Fig. S11C). Besides, our preliminary data shows that palmitoylation may be involved in the regulation of RPS27 at protein level (data not shown). Second, HFD-induced PCa metastasis may also involve other mechanisms in addition to RPS27. For instance, HFD-induced chronic systemic inflammation may also play a role. However, NCG mice were used to exclude the influence of immune alterations.

**Fig. 7:**
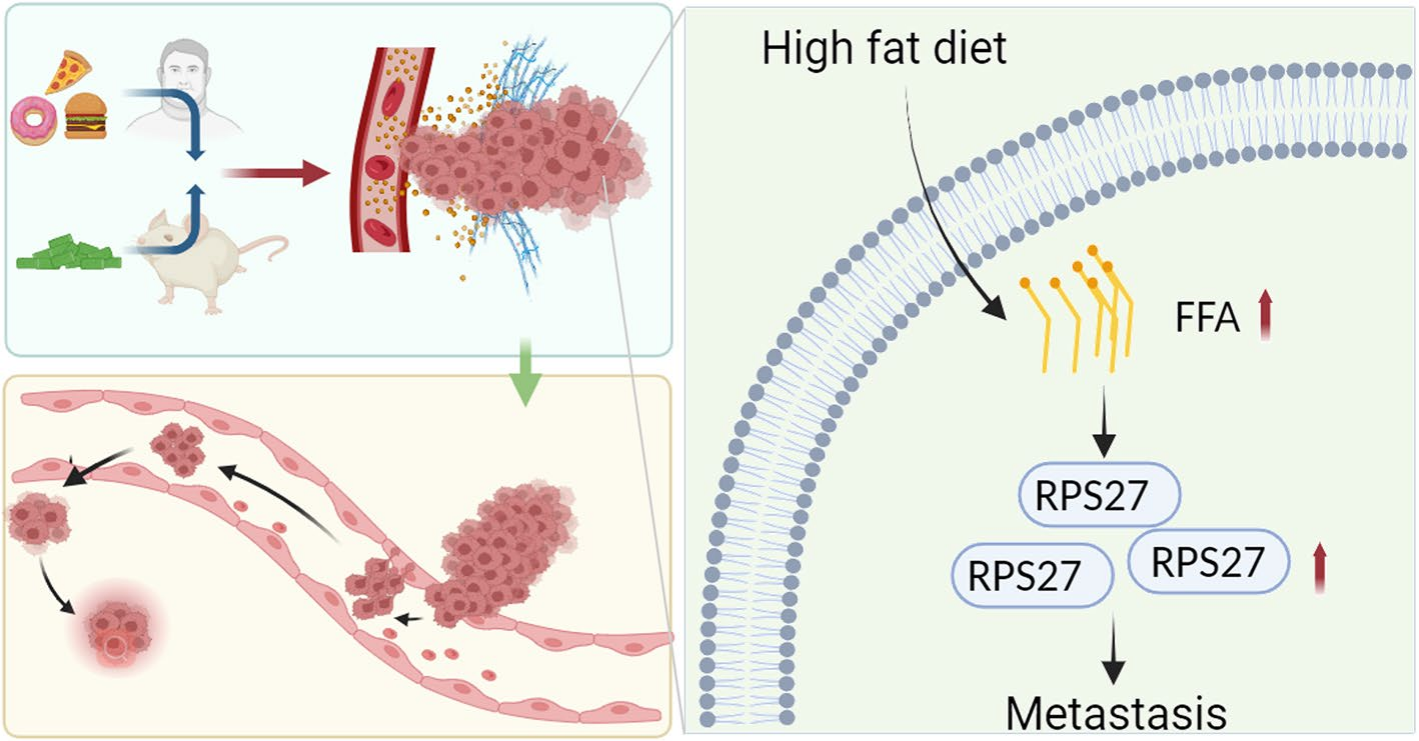
Schematic diagram: High fat diet promotes prostate cancer metastasis via RPS27

In conclusion, our findings demonstrated the role of RPS27 in promoting HFD-induced PCa metastasis. RPS27 may be a potential novel target for the diagnosis and treatment of metastatic PCa (Fig. 7).

### Supplementary Information

**Additional file 1: Supplementary Table 1.** Primers for qRT-PCR. **Supplementary Table 2.** Sequences of shRNAs. **Figure S1.** Images of liver tissue taken from CD and HFD mice (Related to Fig. 1C&1D). (A) 4 weeks; (B) 5 weeks. **Figure S2.** Images of HE staining of the liver tissues (Related to Fig. 1G&1H). **Figure S3.** Protein levels (A) and mRNA expression levels (B) of E-cadherin and vimentin in splenic orthotopic tumors; *P < 0.05; **P < 0.01. **Figure S4.** Western blot of RPS27 in RWPE1, LNCaP, 22RV1, DU145 and PC3 (Related to Fig. 3A). **Figure S5.** Images of migration and invasion assay (related to Fig. 3C&3D). (A) DU145 cells with RPS27 overexpression or control; (B) PC3 cells with RPS27 overexpression or control. **Figure S6.** Images of migration and invasion assay (related to Fig. 3F&3G). (A) DU145 cells with RPS27 knockdown or control; (B) PC3 cells with RPS27 knockdown or control. **Figure S7.** (A) Images of liver tissues taken from EV and RPS27-OE groups (related to Fig. 4A); (B) HE images of the liver tissues from EV and RPS27-OE groups (related to Fig. 4B&4C). **Figure S8.** (A) Images of liver tissues taken from NC and RPS27_sh groups (related to Fig. 4D); (B) HE images of the liver tissues from NC and RPS27_sh groups (related to Fig. 4E&F). **Figure S9.** Images of migration and invasion assay (related to Fig. 3B&3C). (A) Migration; (B) Invasion. **Figure S10.** (A) Images of liver tissues taken from HFD mice inoculated with control or RPS27_sh DU145 cells (related to Fig. 5D); (B) HE images of the liver tissues from HFD and HFD + SH groups (related to Fig. 5E&5F). **Figure S11.** (A) Correlation analysis in PRAD tumor between RPS27 and the transcription factors related to NEFA; Analysis was performed by GEPIA2; Correlation coefficient: Pearson; According to the R value, the order is PPRC1, E2F2, PGC1B, NFKB1, NFKB2, PPARD, PPARA, MYC, YAP, FOXO3, PGC1A, E2F1, PPARG; (B) RPS27 was decreased when E2F2 was knockdown in LNCaP cells; Analysis was performed by KnockTF2.0; (C) Kaplan–Meier analysis of the correlation between disease-free survival in prostate adenocarcinoma patients and E2F2 via GEPIA2; Group cutoff: quartile; Datasets selection: PRAD. **Figure S12.** (A-G) The expression level in EV or RPS27_OE DU145 cells. (A) RPS27; (B) SURF2; (C) FAM83A; (D) AVPI1; (E) TMEM147; (F) TMEM41B; (G) NGRN; (H-N) The expression levels in NC, shRNA_1 and shRNA_2 DU145 cells treated with BSA or NEFA. (H) RPS27; (I) SURF2; (J) FAM83A; (K) AVPI1; (L) TMEM147; (M) TMEM41B; (N) NGRN; **Figure S13.** Kaplan–Meier analysis of the correlation between disease-free survival in prostate adenocarcinoma patients and SURF2, FAM83A, TMEM41B, TMEM147, NGRN and AVPI1 via GEPIA2; Group cutoff: quartile; Datasets selection: PRAD. **Figure S14.** IHC images of RPS27 in liver metastatic tumor. (A) Left: Representative images; Right: IHC scores; Images were captured using the Olympus photomicroscope (Tokyo, Japan), and analyzed using ImageJ (IHC Profiler). The staining intensity was scored as 0 (negative), 1 (positive-low), 2 (positive-median), or 3 (positive-strong). The section score was calculated as the percentage contribution of each staining intensity score multiplied by the score; (B) Related to the right panel of (A); ***P < 0.001. **Figure S15.** Cell growth were detected by CCK8; DU145 or PC3 cells were transfected with RPS27_OE vector or RPS27 siRNAs (si80 + si169), while control group cells were transfected with empty vector or siNC. **Figure S16.** DU145 cells treated with OA (oleic acid) or PA (palmitic acid); Protein levels of RPS27 and GAPDH were detected by western blot.

## Data Availability

The data underlying this article are available in the article and in its online supplementary material.
