# Supplementary material for: High-fat diet promotes prostate cancer metastasis via RPS27
Source: Cancer Metab. 2024 Feb 16;12:6. doi: 10.1186/s40170-024-00333-7 (PMC10870677; doi:10.1186/s40170-024-00333-7)
Supplement: Supplementary file 1 — Additional file 1: Supplementary Table 1. Primers for qRT-PCR. Supplementary Table 2. Sequences of shRNAs. Figure S1. Images of liver tissue taken from CD and HFD mice (Related to Fig. 1C&1D). (A) 4 weeks; (B) 5 weeks. Figure S2. Images of HE staining of the liver tissues (Related to Fig. 1G&1H). Figure S3. Protein levels (A) and mRNA expression levels (B) of E-cadherin and vimentin in splenic orthotopic tumors; *P < 0.05; **P < 0.01. Figure S4. Western blot of RPS27 in RWPE1, LNCaP, 22RV1, DU145 and PC3 (Related to Fig. 3A). Figure S5. Images of migration and invasion assay (related to Fig. 3C&3D). (A) DU145 cells with RPS27 overexpression or control; (B) PC3 cells with RPS27 overexpression or control. Figure S6. Images of migration and invasion assay (related to Fig. 3F&3G). (A) DU145 cells with RPS27 knockdown or control; (B) PC3 cells with RPS27 knockdown or control. Figure S7. (A) Images of liver tissues taken from EV and RPS27-OE groups (related to Fig. 4A); (B) HE images of the liver tissues from EV and RPS27-OE groups (related to Fig. 4B&4C). Figure S8. (A) Images of liver tissues taken from NC and RPS27_sh groups (related to Fig. 4D); (B) HE images of the liver tissues from NC and RPS27_sh groups (related to Fig. 4E&F). Figure S9. Images of migration and invasion assay (related to Fig. 3B&3C). (A) Migration; (B) Invasion. Figure S10. (A) Images of liver tissues taken from HFD mice inoculated with control or RPS27_sh DU145 cells (related to Fig. 5D); (B) HE images of the liver tissues from HFD and HFD + SH groups (related to Fig. 5E&5F). Figure S11. (A) Correlation analysis in PRAD tumor between RPS27 and the transcription factors related to NEFA; Analysis was performed by GEPIA2; Correlation coefficient: Pearson; According to the R value, the order is PPRC1, E2F2, PGC1B, NFKB1, NFKB2, PPARD, PPARA, MYC, YAP, FOXO3, PGC1A, E2F1, PPARG; (B) RPS27 was decreased when E2F2 was knockdown in LNCaP cells; Analysis was performed by KnockTF2.0; (C) Kaplan–Meier analy [file 40170_2024_333_MOESM1_ESM.docx]

**Supplementary Tables**

**Supplementary Table 1. Primers for qRT-PCR**

| Gene | Forward | Reverse |
| --- | --- | --- |
| RPS27 | TGCAGAGCCCCAATTCCTACT | GGCTAAAGACCGTGGTGATTTT |
| GAPDH | GTCTCCTCTGACTTCAACAGCG | ACCACCCTGTTGCTGTAGCCAA |
| CDH1 | TGCCCAGAAAATGAAAAAGG | GTGTATGTGGCAATGCGTTC |
| Vimentin | GAGAACTTTGCCGTTGAAGC | GCTTCCTGTAGGTGGCAATC |

**Supplementary Table 2. Sequences of shRNAs**

|  | sense | Antisense |
| --- | --- | --- |
| shRPS27#1 | CGGTCTTTAGCCATGCACAAA | TTTGTGCATGGCTAAAGACCG |
| shRPS27#2 | CCTACTTCATGGATGTGAAAT | ATTTCACATCCATGAAGTAGG |
| shNC | CAACAAGATGAAGAGCACCAA | TTGGTGCTCTTCATCTTGTTG |

**Supplementary Figures**

**Figure S1**


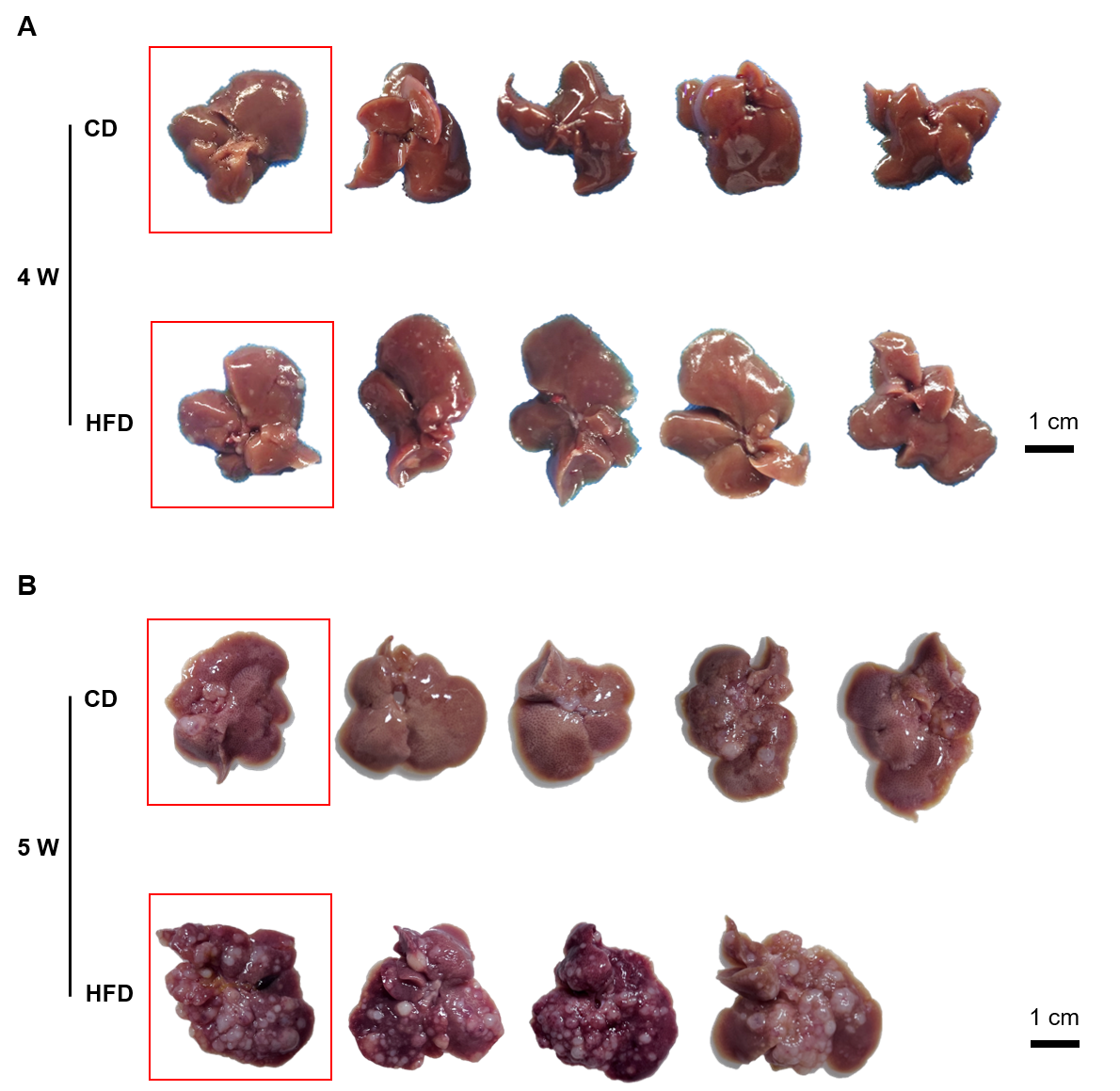


Figure S1| Images of liver tissue taken from CD and HFD mice (Related to Fig. 1C&1D). (A) 4 weeks; (B) 5 weeks.

**Figure S2**


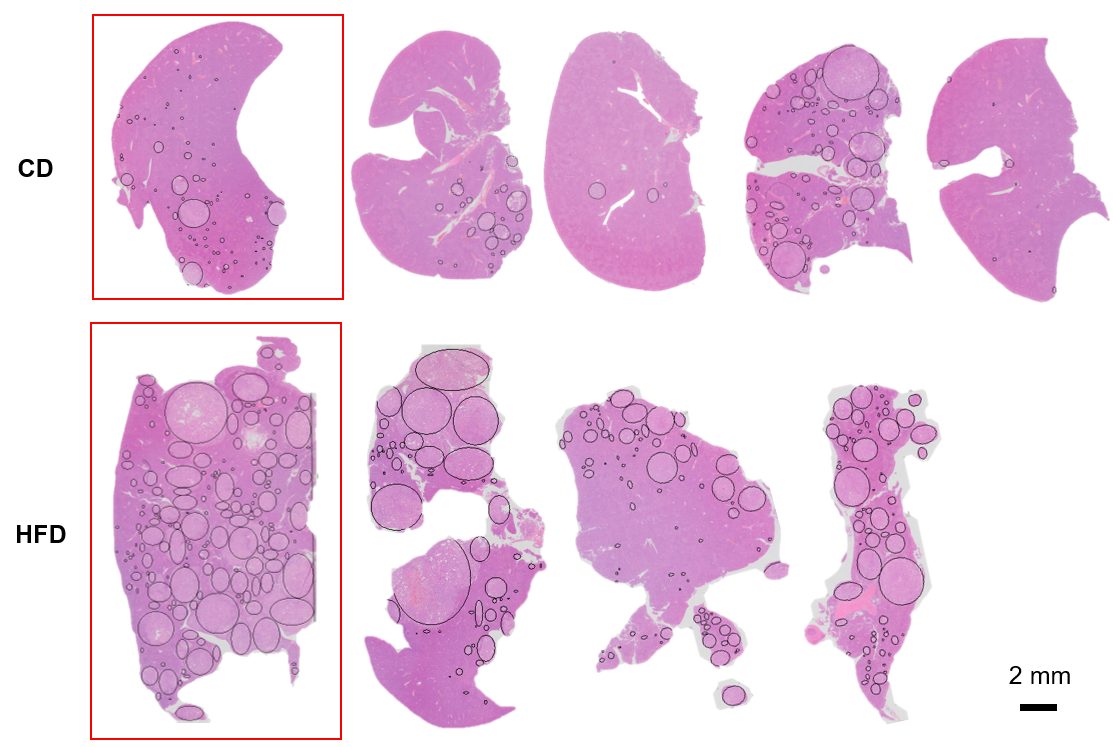


Figure S2| Images of HE staining of the liver tissues (Related to Fig. 1G&1H).

**Figure S3**


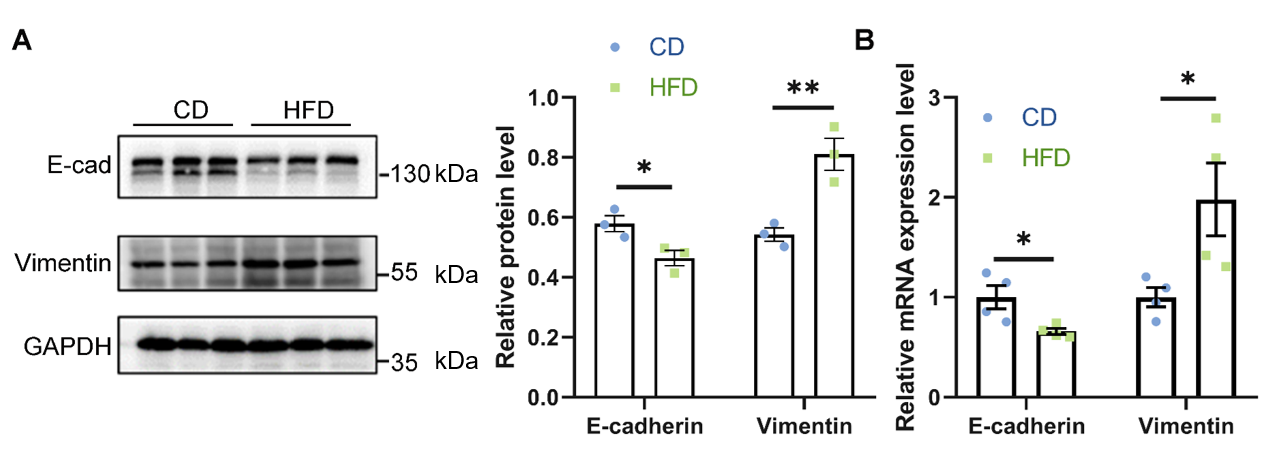


Figure S3| Protein levels (A) and mRNA expression levels (B) of E-cadherin and vimentin in splenic orthotopic tumors; **P* < 0.05; ***P* < 0.01.

**Figure S4**


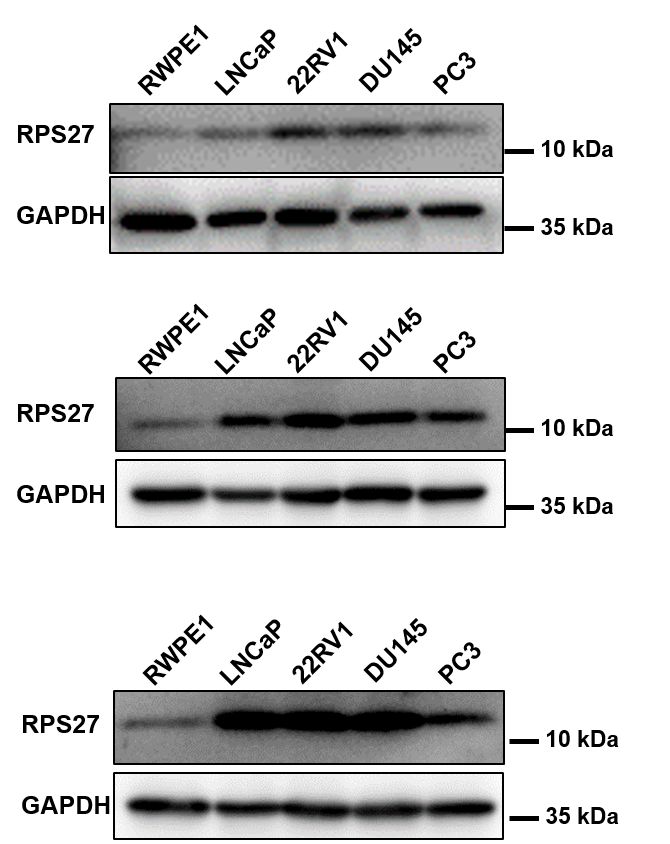


Figure S4| Western blot of RPS27 in RWPE1, LNCaP, 22RV1, DU145 and PC3 (Related to Fig. 3A).

**Figure S5**


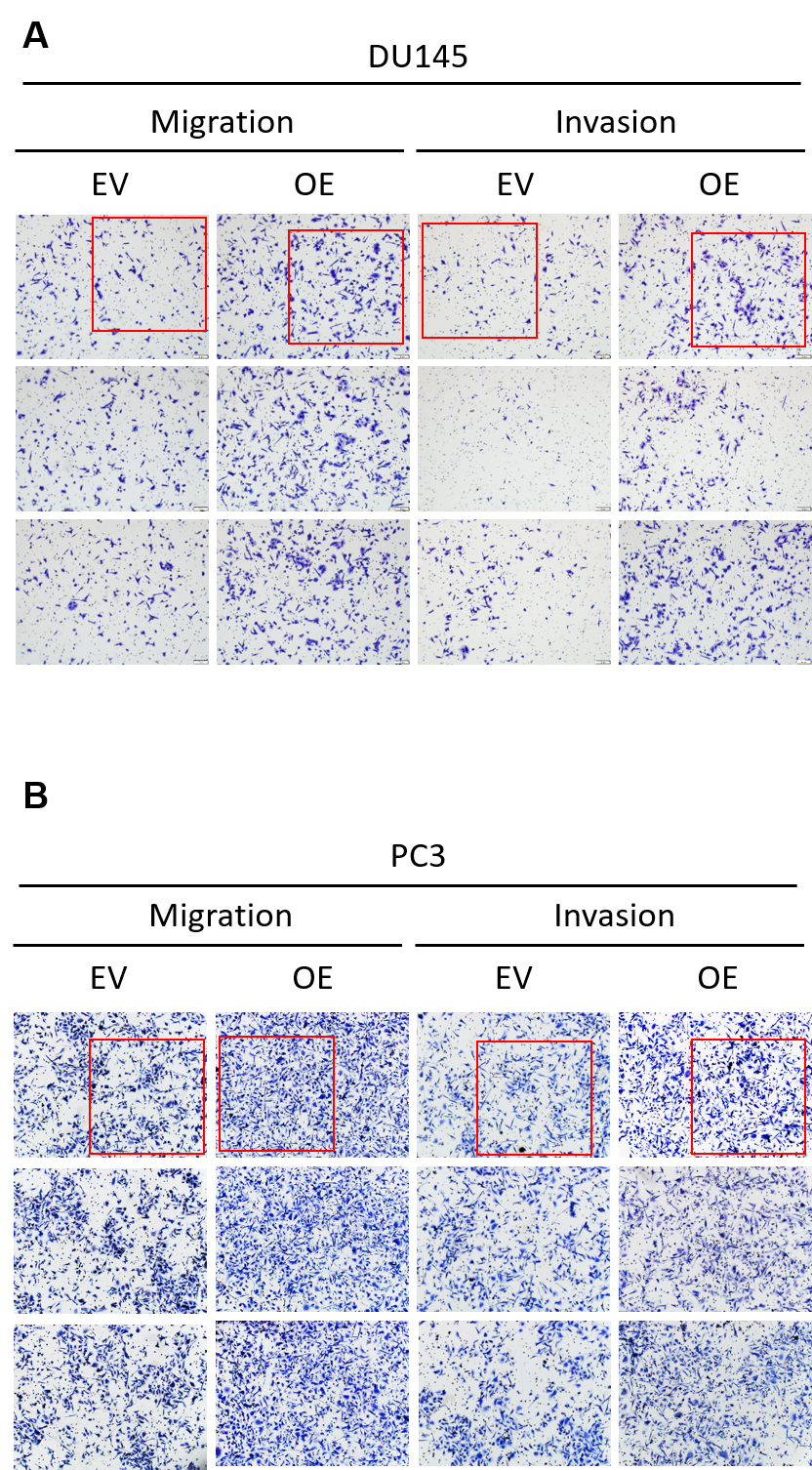

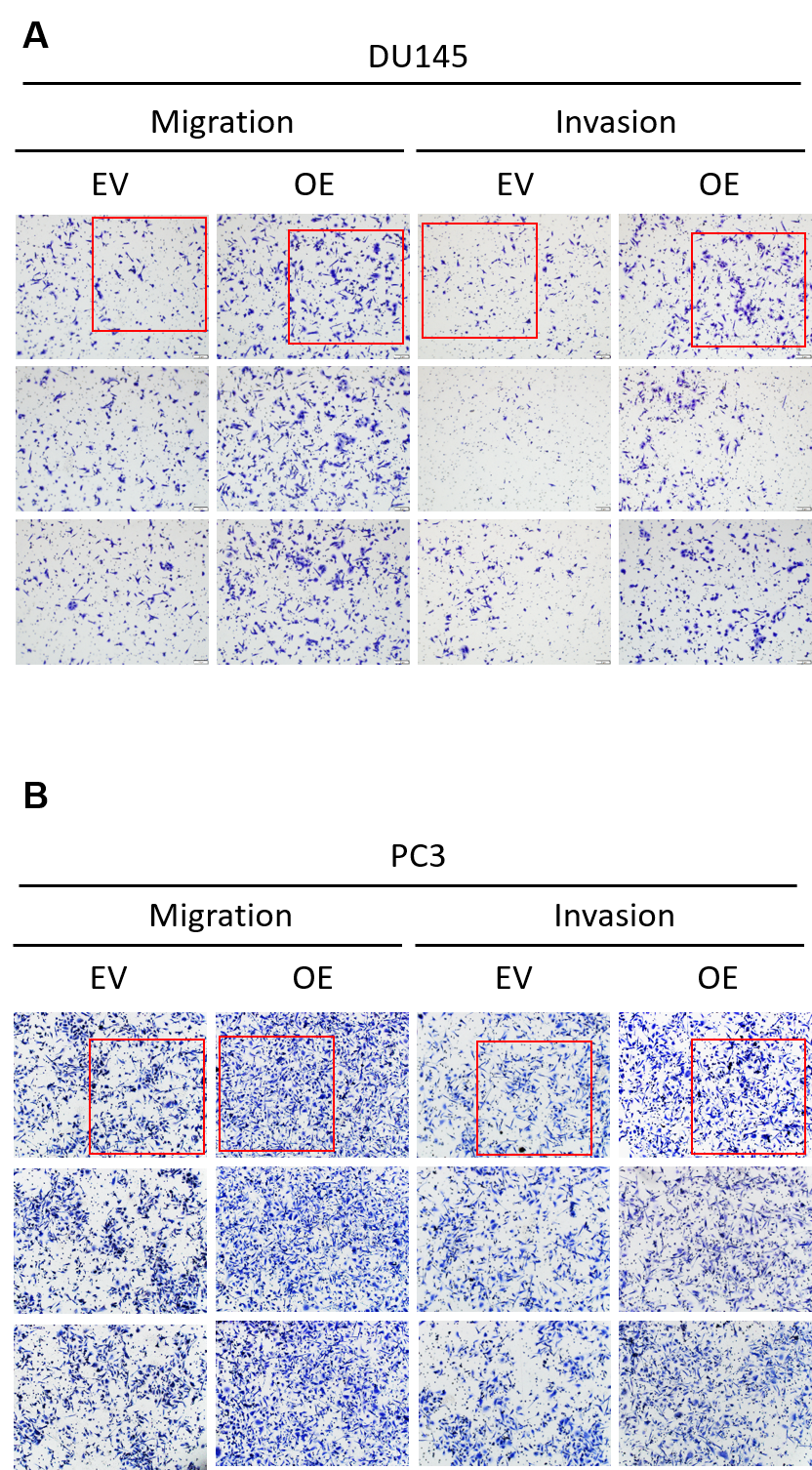


Figure S5| Images of migration and invasion assay (related to Fig. 3C&3D). (A) DU145 cells with RPS27 overexpression or control; (B) PC3 cells with RPS27 overexpression or control.

**Figure S6**


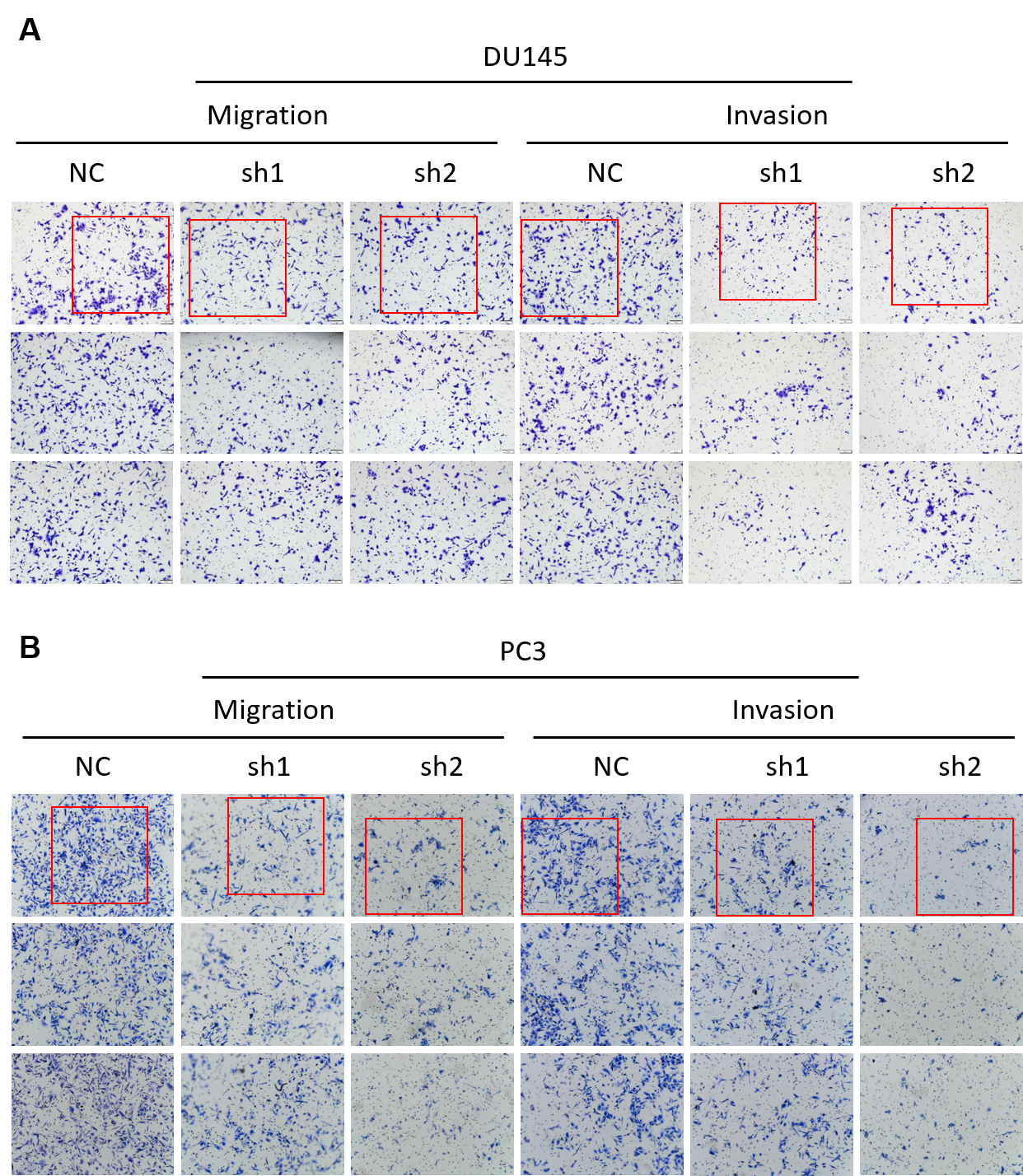


Figure S6| Images of migration and invasion assay (related to Fig. 3F&3G). (A) DU145 cells with RPS27 knockdown or control; (B) PC3 cells with RPS27 knockdown or control.

**Figure S7**


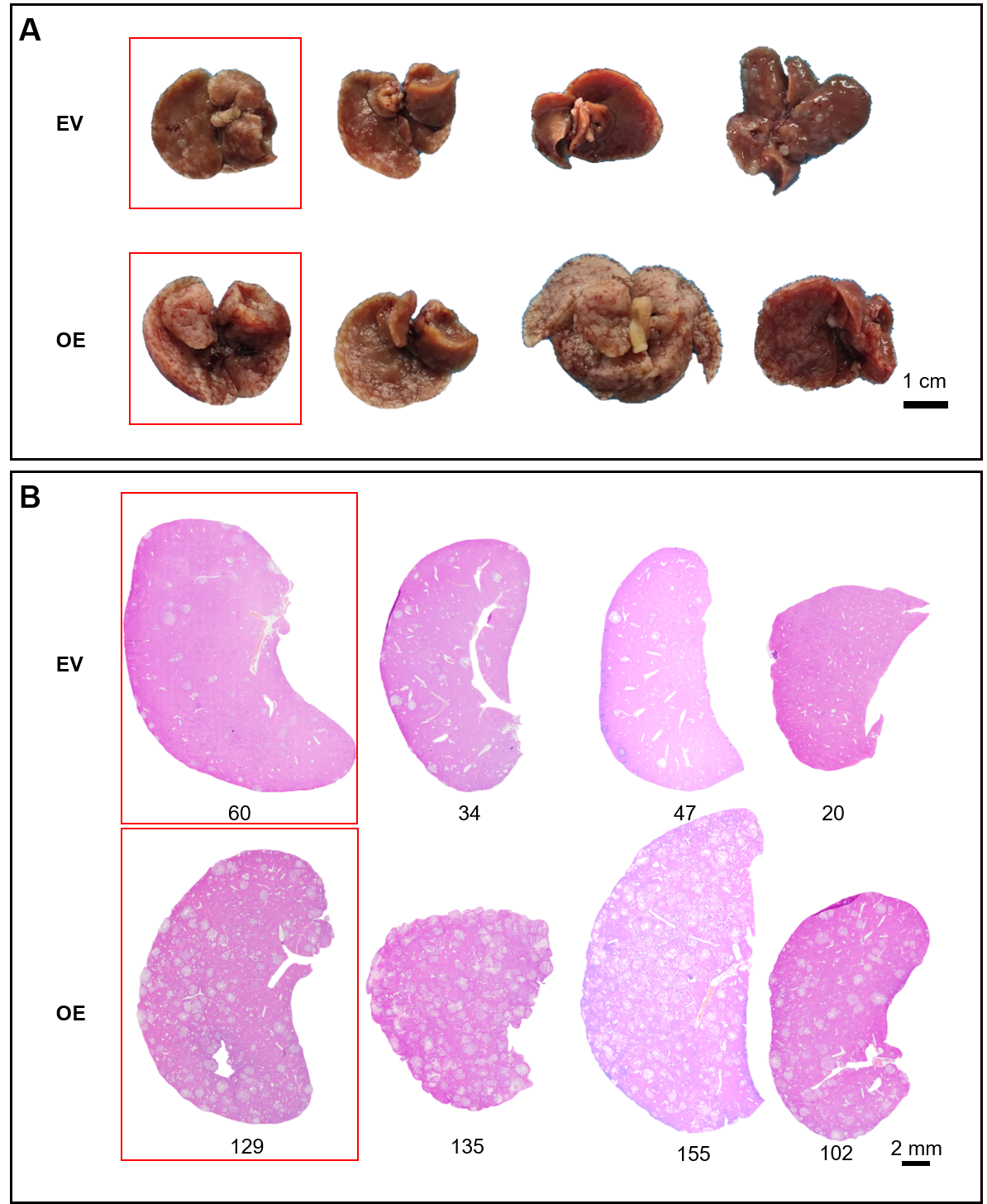


Figure S7| (A) Images of liver tissues taken from EV and RPS27-OE groups (related to Fig. 4A); (B) HE images of the liver tissues from EV and RPS27-OE groups (related to Fig. 4B&4C).

**Figure S8**


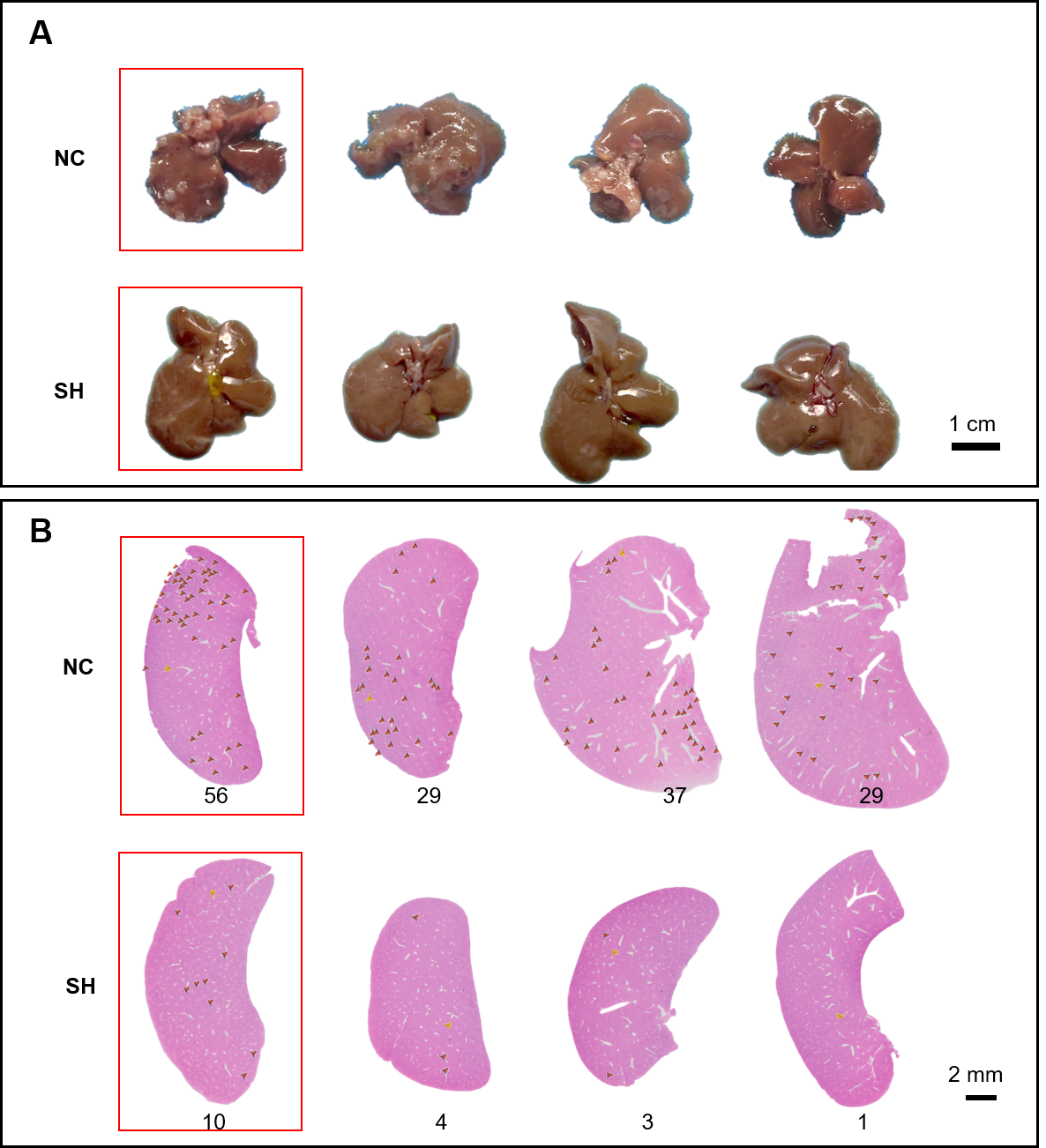


Figure S8| (A) Images of liver tissues taken from NC and RPS27_sh groups (related to Fig. 4D); (B) HE images of the liver tissues from NC and RPS27_sh groups (related to Fig. 4E&4F).

**Figure S9**


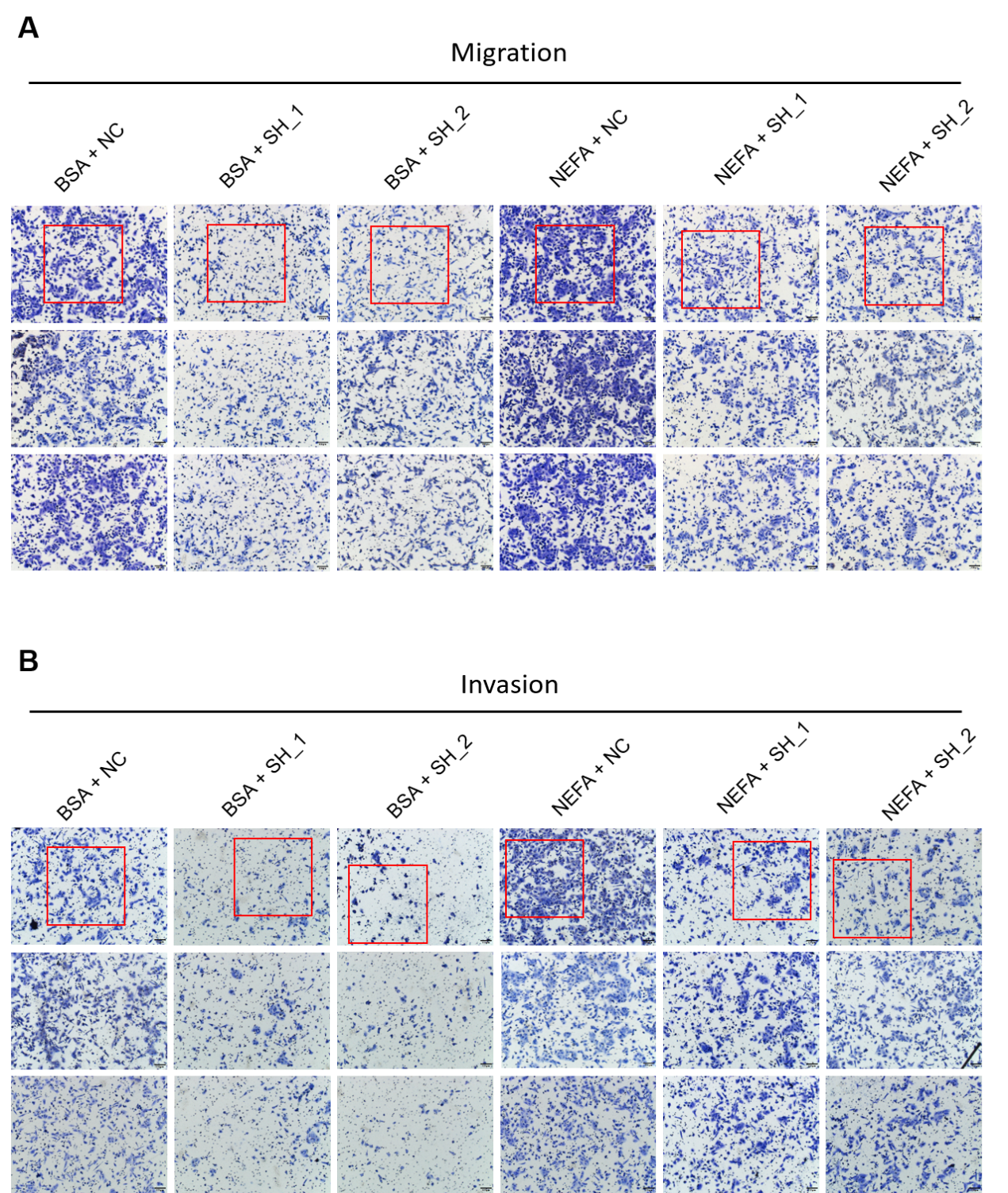


Figure S9| Images of migration and invasion assay (related to Fig. 3B&3C). (A) Migration; (B) Invasion.

**Figure S10**


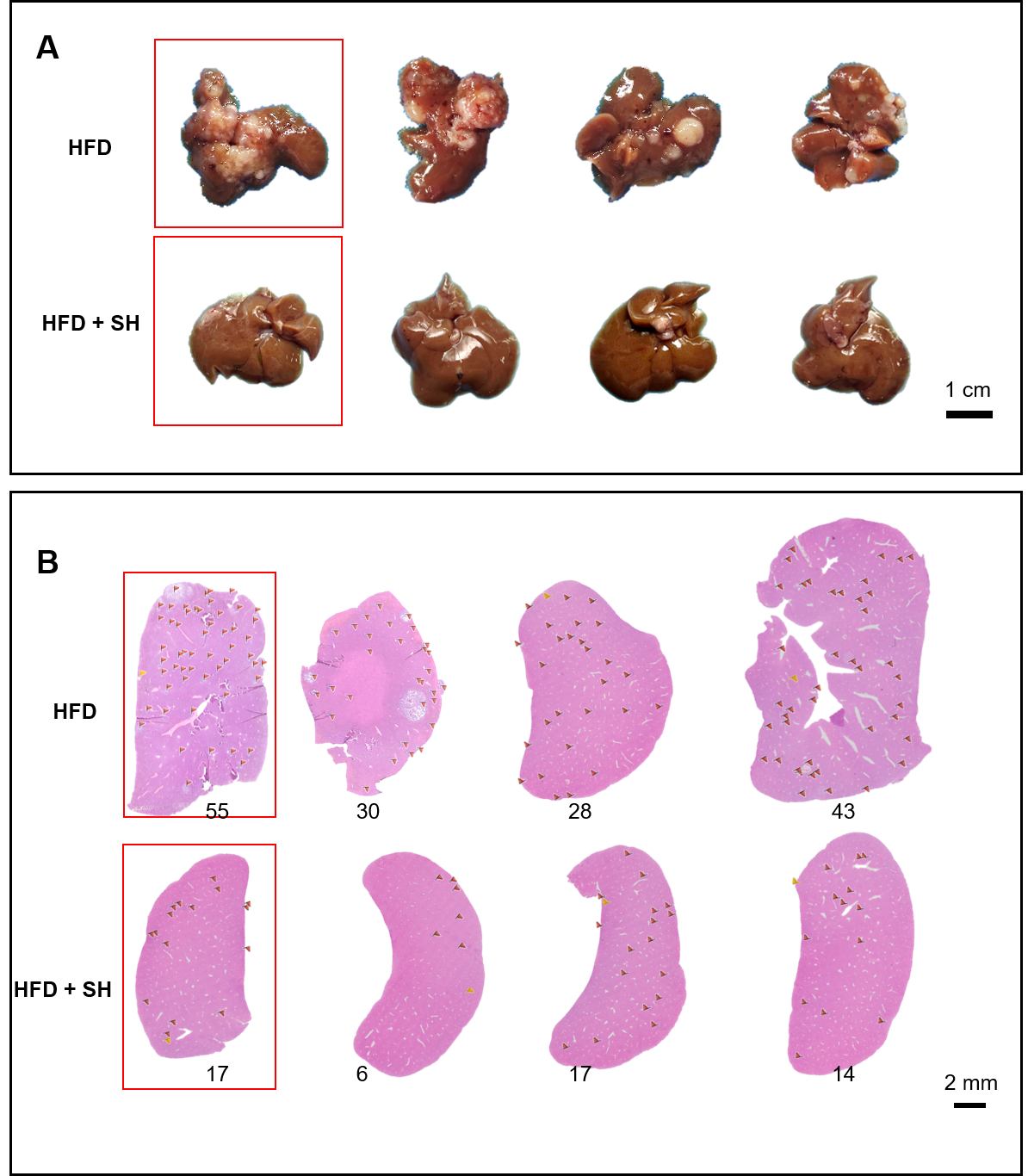


Figure S10| (A) Images of liver tissues taken from HFD mice inoculated with control or RPS27_sh DU145 cells (related to Fig. 5D); (B) HE images of the liver tissues from HFD and HFD+SH groups (related to Fig. 5E&5F).

**Figure S11**


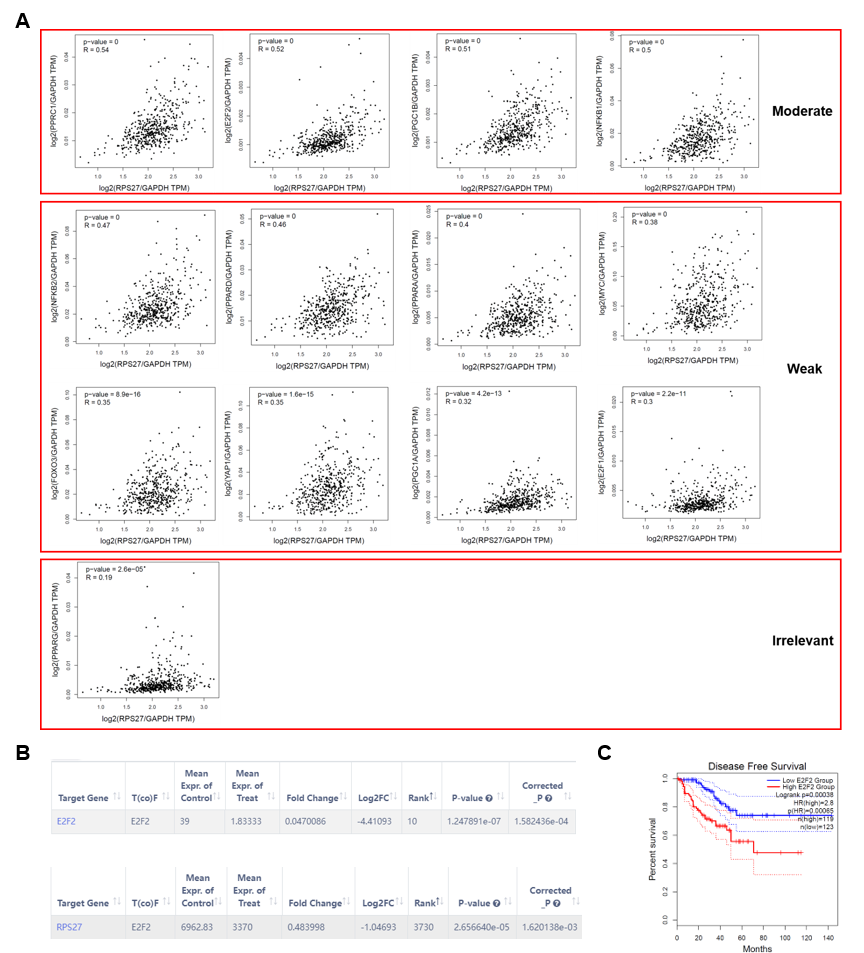


Figure S11| (A) Correlation analysis in PRAD tumor between RPS27 and the transcription factors related to NEFA; Analysis was performed by GEPIA2; Correlation coefficient: Pearson; According to the R value, the order is PPRC1, E2F2, PGC1B, NFKB1, NFKB2, PPARD, PPARA, MYC, YAP, FOXO3, PGC1A, E2F1, PPARG; (B) RPS27 was decreased when E2F2 was knockdown in LNCaP cells; Analysis was performed by KnockTF2.0; (C) Kaplan-Meier analysis of the correlation between disease-free survival in prostate adenocarcinoma patients and E2F2 via GEPIA2; Group cutoff: quartile; Datasets selection: PRAD.

**Figure S12**


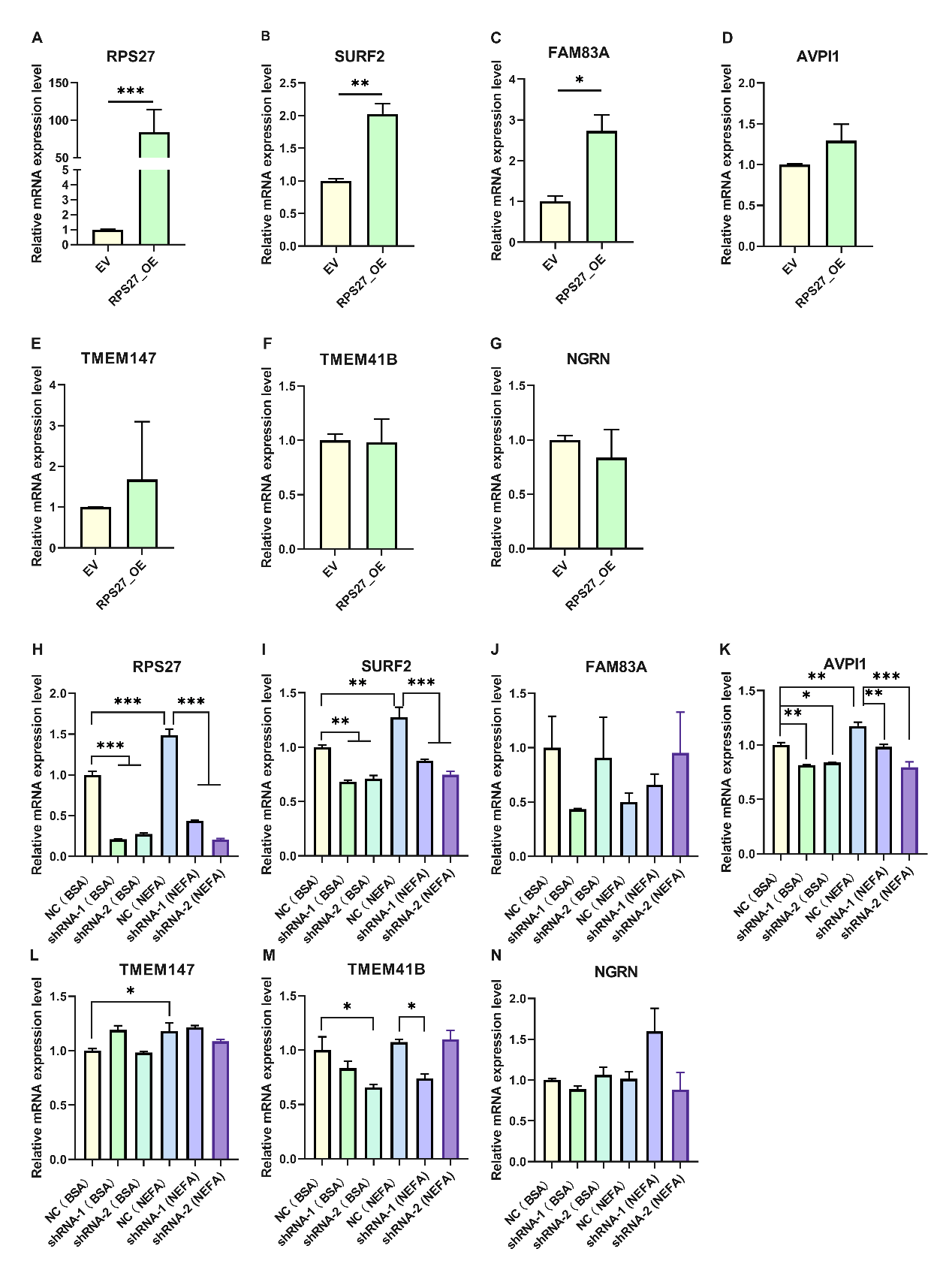


Figure S12| (A-G) The expression level in EV or RPS27_OE DU145 cells. (A) RPS27; (B) SURF2; (C) FAM83A; (D) AVPI1; (E) TMEM147; (F) TMEM41B; (G) NGRN; (H-N) The expression levels in NC, shRNA_1 and shRNA_2 DU145 cells treated with BSA or NEFA. (H) RPS27; (I) SURF2; (J) FAM83A; (K) AVPI1; (L) TMEM147; (M) TMEM41B; (N) NGRN;

**Figure S13**


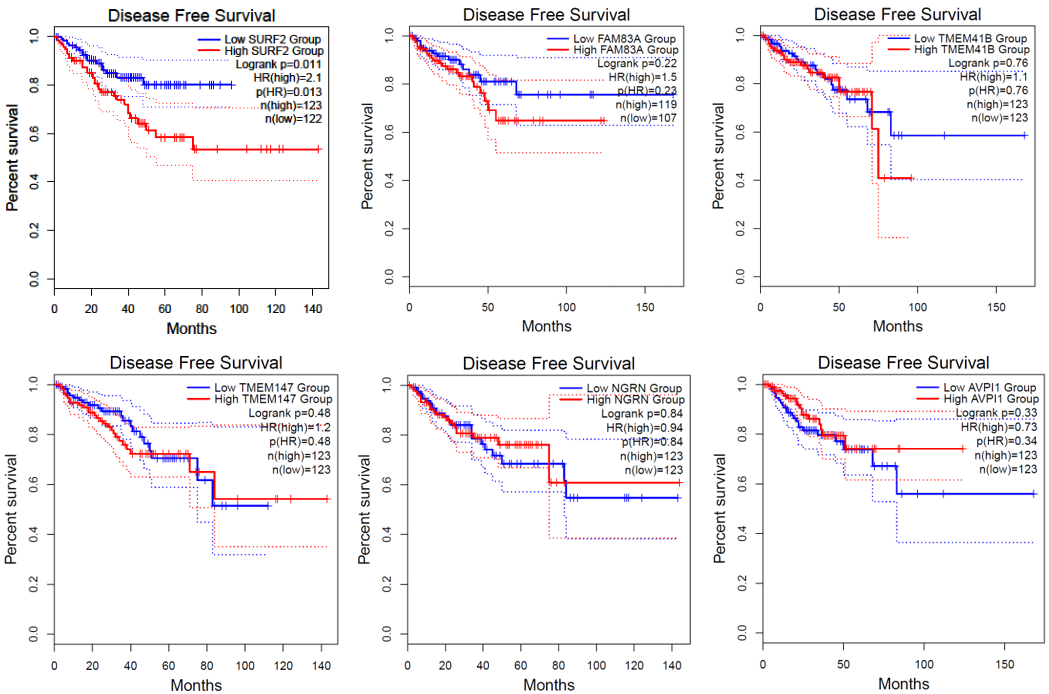


Figure S13| Kaplan-Meier analysis of the correlation between disease-free survival in prostate adenocarcinoma patients and SURF2, FAM83A, TMEM41B, TMEM147, NGRN and AVPI1 via GEPIA2; Group cutoff: quartile; Datasets selection: PRAD.

**Figure S14**

A


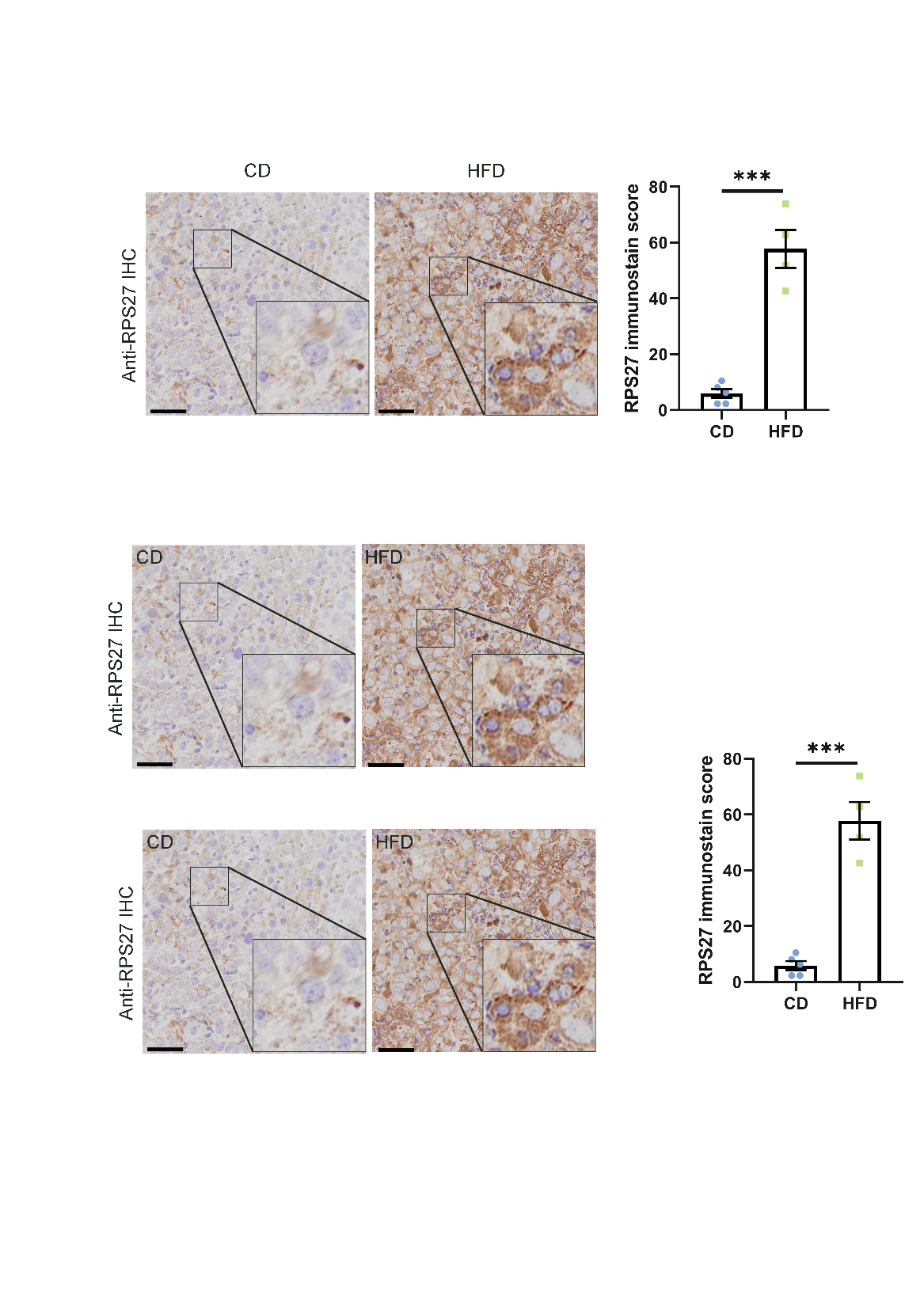


B


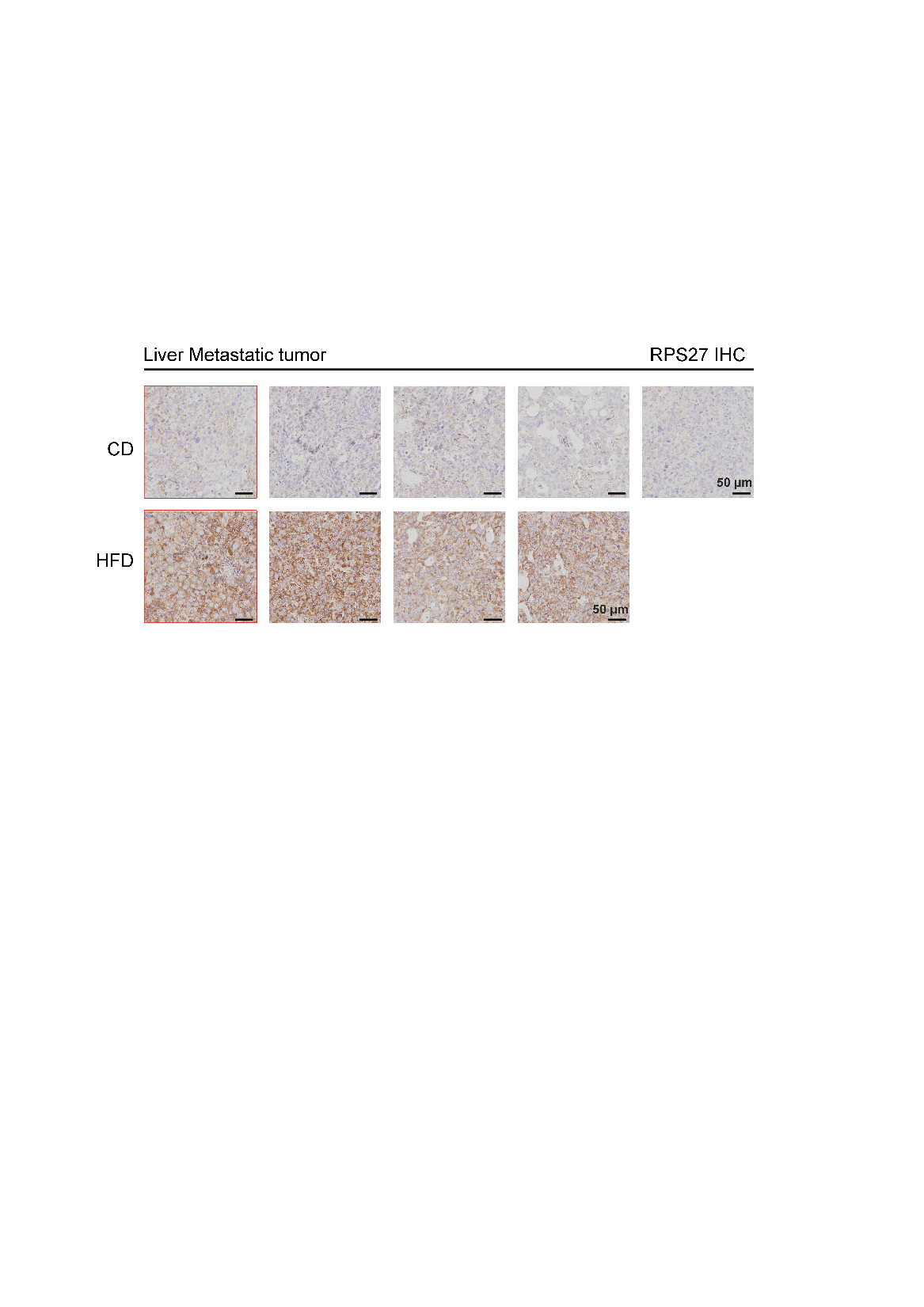


Figure S14| IHC images of RPS27 in liver metastatic tumor. (A) Left: Representative images; Right: IHC scores; Images were captured using the Olympus photomicroscope (Tokyo, Japan), and analyzed using ImageJ (IHC Profiler). The staining intensity was scored as 0 (negative), 1 (positive-low), 2 (positive-median), or 3 (positive-strong). The section score was calculated as the percentage contribution of each staining intensity score multiplied by the score; (B) Related to the right panel of (A); ****P* < 0.001.

**Figure S15**


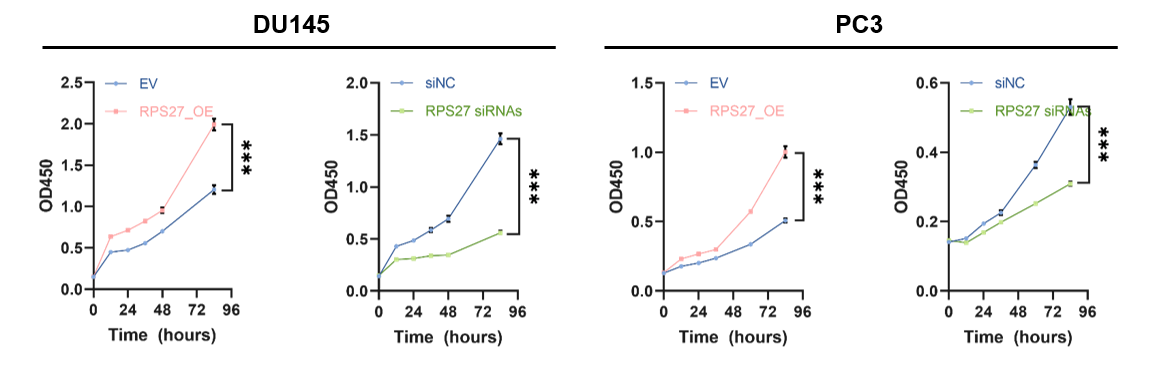


Figure S15| Cell growth were detected by CCK8; DU145 or PC3 cells were transfected with RPS27_OE vector or RPS27 siRNAs (si80 + si169), while control group cells were transfected with empty vector or siNC.

**Figure S16**


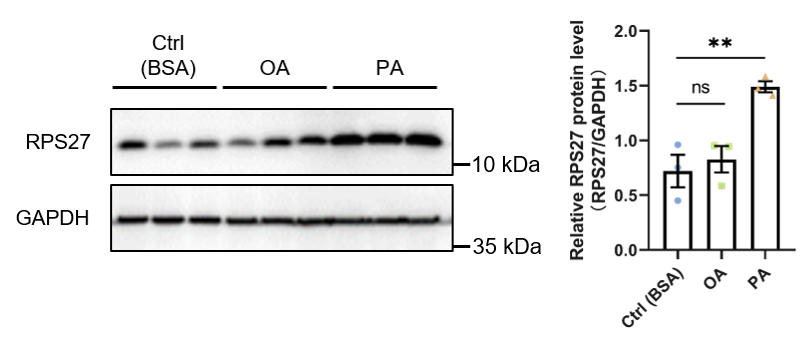


Figure S16| DU145 cells treated with OA (oleic acid) or PA (palmitic acid); Protein levels of RPS27 and GAPDH were detected by western blot.
